# Dating and functional characterization of duplicated genes in the apple (*Malus domestica *Borkh.) by analyzing EST data

**DOI:** 10.1186/1471-2229-10-87

**Published:** 2010-05-14

**Authors:** Javier Sanzol

**Affiliations:** 1 Unidad de Fruticultura, Centro de Investigación y Tecnología Agroalimentaria de Aragón (CITA), Avenida de Montañana 930, 50059 Zaragoza, Spain

## Abstract

**Background:**

Gene duplication is central to genome evolution. In plants, genes can be duplicated through small-scale events and large-scale duplications often involving polyploidy. The apple belongs to the subtribe Pyrinae (Rosaceae), a diverse lineage that originated via allopolyploidization. Both small-scale duplications and polyploidy may have been important mechanisms shaping the genome of this species.

**Results:**

This study evaluates the gene duplication and polyploidy history of the apple by characterizing duplicated genes in this species using EST data. Overall, 68% of the apple genes were clustered into families with a mean copy-number of 4.6. Analysis of the age distribution of gene duplications supported a continuous mode of small-scale duplications, plus two episodes of large-scale duplicates of vastly different ages. The youngest was consistent with the polyploid origin of the Pyrinae 37-48 MYBP, whereas the older may be related to γ-triplication; an ancient hexapolyploidization previously characterized in the four sequenced eurosid genomes and basal to the eurosid-asterid divergence. Duplicated genes were studied for functional diversification with an emphasis on young paralogs; those originated during or after the formation of the Pyrinae lineage. Unequal assignment of single-copy genes and gene families to Gene Ontology categories suggested functional bias in the pattern of gene retention of paralogs. Young paralogs related to signal transduction, metabolism, and energy pathways have been preferentially retained. Non-random retention of duplicated genes seems to have mediated the expansion of gene families, some of which may have substantially increased their members after the origin of the Pyrinae. The joint analysis of over-duplicated functional categories and phylogenies, allowed evaluation of the role of both polyploidy and small-scale duplications during this process. Finally, gene expression analysis indicated that 82% of duplicated genes, including 80% of young paralogs, showed uncorrelated expression profiles, suggesting extensive subfunctionalization and a role of gene duplication in the acquisition of novel patterns of gene expression.

**Conclusions:**

This study reports a genome-wide analysis of the mode of gene duplication in the apple, and provides evidence for its role in genome functional diversification by characterising three major processes: selective retention of paralogs, amplification of gene families, and changes in gene expression.

## Background

Because it provides the raw material for gene diversification and hence the creation of genetic novelty, gene duplication is recognized as a major force in the evolution and adaptation of species [1,2]. The characterization of complete genome sequences has provided evidence that gene duplication is pervasive in eukaryotes and takes place through diverse mechanisms. Gene duplication can range from affecting single genes to whole genomes (polyploidy) [3]. In the simplest situation, small-scale duplications are usually derived from unequal crossing-over or retroposition [4]. Because of its mechanistic peculiarities and frequency, unequal crossing-over produces tandemly arrayed sets of genomic segments (segmental duplications) and is responsible for much of the copy-number variation within species [5-7]. However, segmental duplications can also arise via other mechanisms [4] and can involve the duplication of several genes in a single event by affecting larger chromosomal regions—often tens of kilobases [3]. On the other extreme, whole-genome duplications (polyploidy) are less frequent but have a major impact in the generation of genetic diversity because all the genes in the genome are duplicated simultaneously [4].

Although small-scale duplications or large segmental duplications seem to be a phenomena common to all eukaryotes, polyploidy appears to be more restricted to specific lineages and is particularly well recognized among flowering plants [8]. It has been estimated that up to 70% of all angiosperms may have experienced at least one episode of polyploidy in their evolutionary history [8]. Moreover, the recent analyses of the complete sequence of four eurosid genomes has provided unexpected evidence of an ancient genome duplication that occurred early in the evolution of these species, suggesting a polyploid origin for the majority of eudicots [9,10]. Overall, duplicated genes represent a large proportion of the gene complement of plant genomes. For example, up to 80% of the *Arabidopsis *and more than half of the poplar and rice genes are grouped into gene families [11-13]. Accumulating data also suggest that gene families are dynamic entities subject to expansion/contraction and creation/extinction events that are in close association with the diversification and adaptation of plant lineages [14-16].

The prevailing theory predicts gene loss or functional diversification as the main fate for one of two copies of a pair of duplicated genes [17]. Gene loss results from partial or complete deletion, pseudogenization, or gene silencing. Experimental observations have revealed that gene loss is a highly regulated process that is influenced both by the time and origin of the gene duplication. Genome-scale analyses suggest that marked differences exist regarding the functions of duplicate genes, depending on whether they originated from small-scale or large-scale duplications [18]. Similarly, the patterns of gene retention seem to differ among taxonomic categories, indicating different modes of gene duplication within different plant groups [19].

When the two copies of a duplicated gene are retained in a functional state, they can diversify by partitioning the original function (subfunctionalization) or by evolving a new function (neofunctionalization). Of these two processes, subfunctionalization processes, in which each duplicate gene develops a distinct expression pattern, seems to be the most common phenomena and takes place early after gene duplication [20]. On the other hand, neofunctionalization is the result of diversification over the long term [21]. Nevertheless, two paralogs can selectively retain identical functions and expression patterns if, for example, higher levels of gene expression can be achieved through increased gene dosage [22].

The apple (*Malus domestica *Borkh.) is one of the most important fruit crops grown in temperate climates and is widely appreciated by consumers worldwide. Together with other fruit species such as pears, quince, and loquat, apples are grouped into the subtribe Pyrinae (formerly subfamily Maloideae) within the family Rosaceae, and have a pome-type fruit and chromosome number *x *= 17 as the main distinctive characteristics. This high base chromosome number compared with other members of the Rosaceae family (typically with *x *= 7, 8, or 9) was recognized early as the result of polyploidy. Sax [23] proposed the hypothesis of an allotetraploid origin of the subtribe Pyrinae through wide-hybridization between progenitors with *x *= 8 and *x *= 9. Moreover, he suggested that these progenitors may have been related to extant members of the Rosaceae family belonging to the Amigdaloideae (*x *= 8) and Spiroideae (*x *= 9) lineages. A more recent molecular phylogenetic analysis by Evans and Campbell [24] supported the allotetraploid origin of the subtribe Pyrinae. However, their data contradicted the wide-hybridization hypothesis; instead these authors suggested that the founding hybridization involved two closely related species ancestral to the genus *Gillenia *(*x *= 9), followed by loss of one pair of chromosomes [24]. Despite the early identification of this plant lineage as originating from polyploidy, which suggests a prominent role for gene duplication in its diversification, little is known about the dynamics and mode of gene duplication operating within this taxonomic group.

Collections of expressed sequence tags (ESTs) are providing plant scientists with a valuable source of data for the large-scale characterization of duplicated genes in non-sequenced genomes [12,19,25-27]. In the apple, different sequencing projects have produced large collections of ESTs from libraries covering a variety of genotypes, tissues, and experimental conditions [28]. Two initiatives in particular [29,30] account for the majority of the EST resources currently available in the species. 'GoldRush' and 'Royal Gala' are the two cultivars in which much sequencing effort has been invested. Mining these EST collections has allowed considerable advancement in the development of DNA-based markers, i.e., simple sequence repeat (SSR) and single nucleotide polymorphisms (SNP). In addition, functional annotation and digital expression analysis of these EST libraries provide efficient tools for gene discovery [31] and comparative genomics [30]. The aim of the present study was to date and characterize duplicated genes in the apple using these publicly available EST resources. Because the apple is a highly polymorphic species, sequence collections obtained from a single cultivar were considered a more suitable means to reduce the possibility of false characterization of allelic variants as recently evolved duplicated genes. Also, because changes in gene expression are important outcomes of the functional diversification of duplicated genes, the selection of a dataset composed exclusively of non-normalized libraries allows one to take advantage of *in silico *methods for monitoring gene expression [32]. Based on these considerations, the set of non-normalized libraries obtained by Newcomb et al. [29] for the cultivar Royal Gala, containing ~120,000 high-quality ESTs and covering a range of different tissues, was considered a suitable dataset for this investigation.

The present study reports the characterization of duplicated genes and their derived gene families in the apple using EST sequence collections. I dated the age of gene duplications using synonymous site substitutions, to identify the major events that contributed to the formation of paralogous genes in the species. This approach allowed characterization of a continuous mode of small-scale duplications and two putative episodes of polyploidy. The imprint left by this mode of gene duplication was studied and suggested that the selective retention of paralogs, the amplification of gene families, and the acquisition of novel patterns of gene expression by duplicated genes have been important mechanisms shaping the apple genome.

## Results and Discussion

### Construction of unigenes

Thirty-three publicly available EST libraries containing 119,177 sequences from the apple cultivar Royal Gala were used to construct a unigene dataset of tentative non-redundant sequences (see Additional file 1). The EST libraries represented 14 different tissues and/or developmental stages (see Additional file 2). ESTs were assembled yielding 13,168 contigs and 20,044 singletons with average lengths of 744 and 411 nucleotides, respectively (Table 1). Overall, these parameters were very similar to those reported by Newcomb et al. [29], whose dataset mainly included the libraries used for this study. In an attempt to prevent the formation of chimeras derived from the assembly of sequences belonging to close paralogous genes, the assembly project reported here involved slightly more stringent conditions during clustering. However, neither the proportion of consensus sequences over singletons nor the resulting mean sequence lengths differed substantially from the results reported by Newcomb et al. [29], suggesting that the conservative approach I used did not affect the efficacy of the assembly procedure.

**Table 1:** EST clustering and assembly data

	Number of sequences	Mean length
ESTs used for assembly	116,879	471
ESTs in clusters	96,836	483
Contigs	13,168	744
Singletons (non clustered ESTs)	20,043	411
Unigenes (contigs + singletons)	33,211	543

Unigenes were used for BLASTX homology searches against the plantRefSeq database. A total of 19,732 sequences matched known genes, representing ~60% of the total unigene set. The remaining sequences (~40%), were likely candidates for apple-specific genes, genes with uncharacterized open reading frames, transcripts from non-coding genes, or transcribed genomic regions corresponding to mobile elements or other repetitive DNA, and were discarded from further analysis. A further selection of this initial dataset of putative protein-coding genes was made by discarding transcripts with open reading frames shorter than 300 nucleotides. After removing those unigenes matching (retro)transposon-related proteins and the sequences putatively corresponding to the same transcript (see Methods), the resulting dataset consisted of 13,598 non-redundant protein-coding genes (Table 2).

**Table 2:** Unigene analysis statistics

Dataset	Number of unigenes
Unigenes with BLASTX match (E-cutoff 1e-10)	19,732
Selected set of protein-coding genes	13,598
Genes in families	9,339
Mean number of genes per familly	4.6
Single-copy genes (singlets)	4,259

### The age distribution of gene duplications

**Figure 1:**
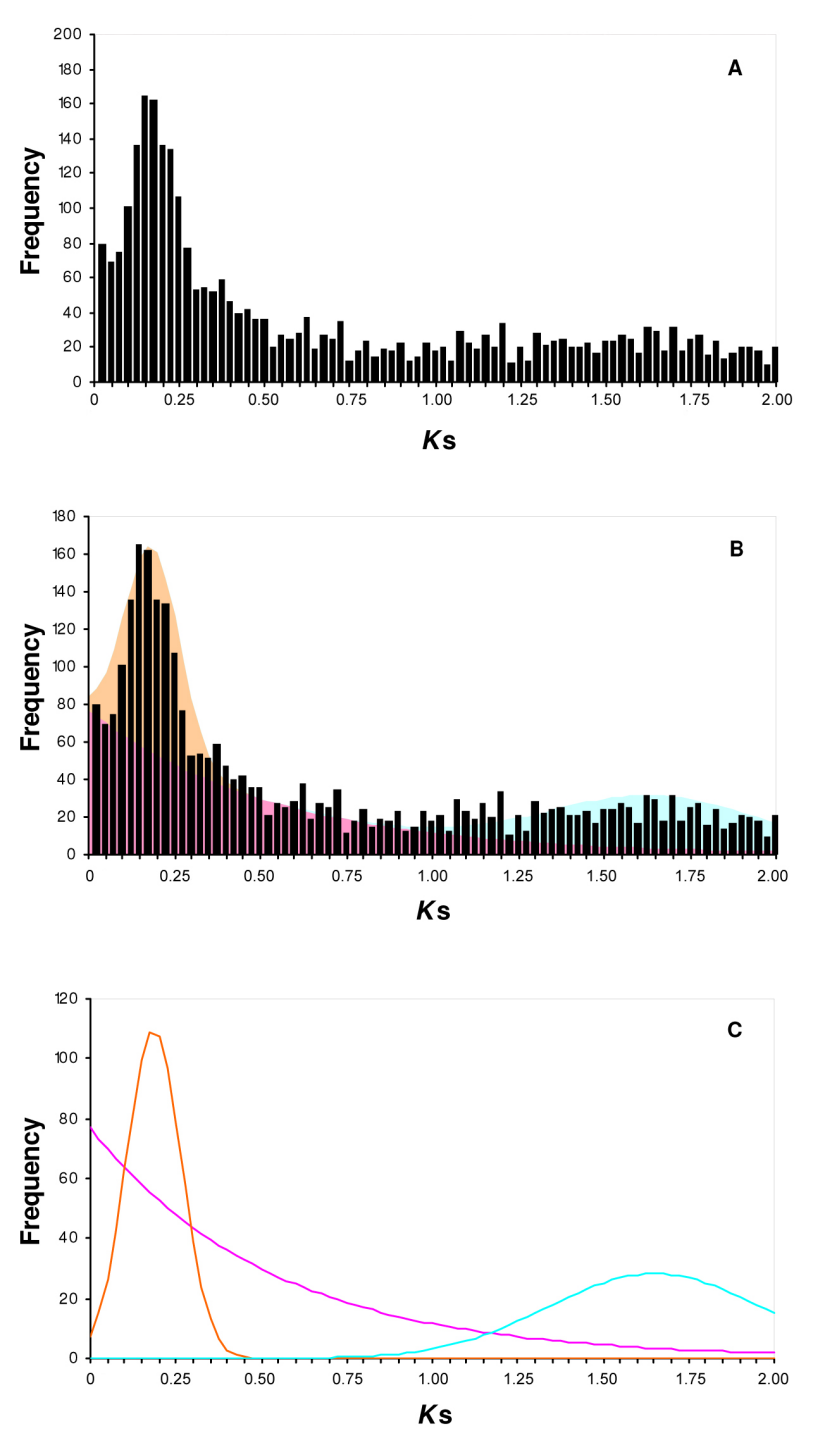
**Age distribution of gene duplications in the apple**. **(A) **Frequency distribution of apple gene duplications as a function of the *K*_s _distance (synonymous substitutions per synonymous site). **(B) **Superimposed distributions of the observed apple duplications (bars) and a cumulative function derived from an exponential (pink) decay of small-scale duplications and two normals (orange and blue) corresponding to two putative independent episodes of polyploidy. **(C) **Deduced distributions representing the continuous mode of small-scale duplications (pink), the Pyrinae polyploidization (orange), and the γ-triplication event (blue).

Pairs of duplicated genes (paralogs) were characterized based on protein sequence similarity. In the collection of 13,598 protein-coding genes, 9,339 (~68%) were found to have at least one paralog, whereas 4,259 (~32%) did not match with any other sequence in the dataset and were considered to be single-copy genes (singlets). The ages of the gene duplications were estimated by computing the *K*_s _distance (synonymous substitutions/synonymous site) between pairs of paralog sequences. Figure 1a shows the distribution of the gene duplication frequencies as a function of the *K*_s _distance.

In an attempt to understand the events shaping the distribution of gene duplications in the apple, the fit of the distribution was evaluated to a mixture of densities involving an exponential distribution representing the continuous mode of small-scale duplications and several normal distributions representing putative large-scale duplication events. The best significant fit was found with a mixture of exponential decay of small-scale duplications with a death rate of 1.892 and two normal distributions with median *K*_s _values of 0.185 and 1.650 respectively, which were attributable to two putative episodes of whole-genome duplication (Figure 1b). The presence of the youngest peak was consistent with the allopolyploid origin of the Pyrinae genome, and supports on a genomic scale this polyploidization event that was initially inferred based of cytological data [23] and later confirmed following genetic marker analysis [33]. The sharp peak created by this putative most recent episode of genome duplication allowed estimation of the time of divergence of the two species that originated the ancestor of the subtribe Pyrinae at a median *K*_s _= 0.185. This estimate should be somewhat older than the age of the polyploidization event itself, because an interspecific fusion has been generally accepted for the origin of this lineage [24]. This *K*_s _age seems to reflect a slow rate of molecular evolution [24]. In fact, if the clock rates of synonymous substitutions frequently used for other eudicots [34,35] are assumed, the time of the Pyrinae origin could not be dated earlier than 6-15 million years before present (MYBP), whereas according to fossil data, this lineage appears to extend back to the Middle Eocene [36] at 37-48 MYBP. This discrepancy could at least partially be explained by the slow pace of molecular evolution observed for woody plants, which is related to the lengthy generation time of these species [37]. Analysis of the Salicoid polyploidization of the poplar genome by Tuscan et al. [38] showed similar slow rates of molecular evolution. In that study the authors argued that the long persistence of a poplar genotype as a clone would favour the recurrent contribution of "ancient gametes" by very old individuals, reducing the rates of mutation. A similar scenario could be envisioned for the evolution of the apple due to the woody nature of this species and frequent vegetative reproduction.

The analysis of the mixture densities also identified an older wave of gene duplications with median *K*_s _= 1.650 (Figure 1). Interestingly, although the signal observed for this putative second episode of large-scale duplications was weaker and showed a range of variation much wider than that observed for the younger peak, its median age and variance were consistent with the values estimated for the γ-triplication in other species (Table 3). This ancient episode of hexapolyploidization has been characterized in the four eurosid genomes sequenced to date, and its origin could be traced back to the common ancestor of all eudicots if it is also confirmed in species in the Asterid group [9,10,19,39,40].

**Table 3:** Comparative analysis of the γ-triplication in different species

Species	Source of data	Median	Variance	Reference
*Arabidopsis*	Whole genome	2.00	0.20	Tang et al. 2008
Poplar	Whole genome	1.54	0.24	Tang et al. 2008
Papaya	Whole genome	1.76	0.32	Tang et al. 2008
Grape	Whole genome	1.54	0.16	Tang et al. 2008
Compositae (18 species)^a^	EST	1.20-2.00	-	Barker et al. 2008
Apple	EST	1.65	0.31	This study

^a^Ancillary peak observed in 18 Compositae species on the *K*s range indicated and that was related by the authors

Median and variance estimates for the age (*K*_s_) distribution of paralogs derived from the *γ*-triplication in different species

The normal distribution of gene duplications corresponding to the younger peak (*K*_s _= 0.185; Figure 1c) predicted that ~865 pairs of duplicated genes showing *K*_s _ages younger than 0.4 might be related to this episode of polyploidization (Figure 1c). It is likely however, that this value is an underestimate of the actual number of retained genes in the dataset, because rates of silent mutations among simultaneously duplicated genes often vary greatly [41,42]: up to 14-fold in *Arabidopsis *[41]. It is therefore expected that a proportion of the apple paralogs created during this episode of polyploidy will have higher computed *K*_s _distances. Despite this expected variation, it is relevant for the purpose of this study that the group of paralogous genes having *K*_s _≤ 0.4 likely contains the majority of the duplicates formed during the Pyrinae polyploidization. Similarly, as this episode of polyploidization marks the onset of the Pyrinae lineage, paralogs with *K*_s _≤ 0.4 should also contain the majority of the small-scale duplications that arose after the formation of the Pyrinae ancestor. This group of duplicated genes will be considered paralogs specific to the genome of the Pyrinae group or eventually specific to the apple genome, and will be referred to collectively as young duplicates in further analysis.

### Characterization and size distribution of gene families

**Figure 2:**
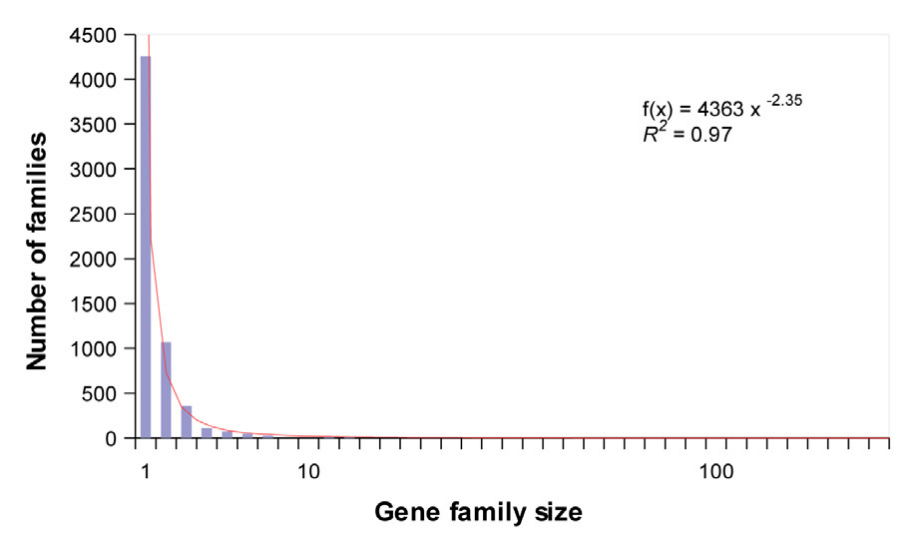
**Size distribution of apple gene families**. Distribution of gene family sizes in the apple estimated by single-linkage clustering of BLASTP sequence similarity searches. Gray line: best fit of the distribution to a power-law with the equation f(*x*) = 4363*x*^-2.35 ^(*R*^*2 *^= 0.97).

Single-linkage clustering grouped the set of paralogs into 2018 gene families yielding a mean number of 4.6 genes per family (Table 2; Figure 2). Gene family size varied substantially however, with clusters ranging from two members to 233 members for the gene superfamily of plant receptor-protein kinases (Table 4). To better understand the variation in gene family size in the apple, the frequency of each size-class was calculated and showed a distribution that closely approximated a power-law with the equation: 4363*x*^-2.35 ^(Figure 2). According to this type of distribution, a few families have many members, whereas many families have only a few members. For the apple in particular, the number of gene families is expected to be 4.29 times lower as the size of the gene family is duplicated. As new species are being analyzed, power-law distributions of gene family sizes seem to be ubiquitous across all organisms, with few exceptions [43,44]. This suggests that a common evolutionary force controls the dynamics of gene family size. Selection for useful functions along with versatility to work in a variety of molecular functions, are likely key aspects for the dominance of specific gene families in genomes [45]. In the apple, the largest gene families (Table 4) are protein kinases, which include the protein receptor kinases and the mitogen-activated protein kinases, the cytochrome P450 gene superfamily and the NBS-LRR resistance proteins. All these, are also among some of the most populated gene families in other plant species [38,46,47]. The observation that the above families have evolved to participate in a wide variety of biological functions [48-51] is consistent with the hypothesis that molecular versatility is important in determining the dominance of some gene families in genomes. Most remarkably, genes encoding protein receptor kinases, mitogen-activated protein kinases, and NBS-LRR resistant proteins, function in the transduction of multiple external signals [48,49,51]. The sessile nature of plants may have favoured the expansion of those families involved in diversifying the capacity to adapt to the environment.

**Table 4:** Most populated gene families in the apple

Gene family	Gene-copy number
Protein receptor kinases (PRK)	233
Mitogen-activated protein kinases (MAPK)	136
Cytochrome P450	118
NBS-LRR resistance proteins	104
Glycosyltransferase family 1	71
Ras-related GTP-binding proteins	64
Leucine-rich repeat proteins (non-kinase domain)	59
Hypothetical; nucleic acid-binding protein familly	56
Oxidoreductases; Fe(II) oxigenase family	53
Phosphoprotein phosphatases; ser/thr phosphatases	50
E2 ubiquitin-conjugating enzyme	49
MYB transcription factors	49
26s proteasome aaa-atpase subunit	48
Eukaryotic initiation factor 4A (eIF4A/eIF4A-like)	47
PP2C-type Phosphatases	45
WD-repeat protein superfamily	41
Hypothetical	39
Acyltransferase	37
Hypothetical ATP-binding	36
Short-chain dehydrogenases/reductases	36

Twenty largest apple gene families and their estimated gene-copy number in the EST dataset.

**Figure 3:**
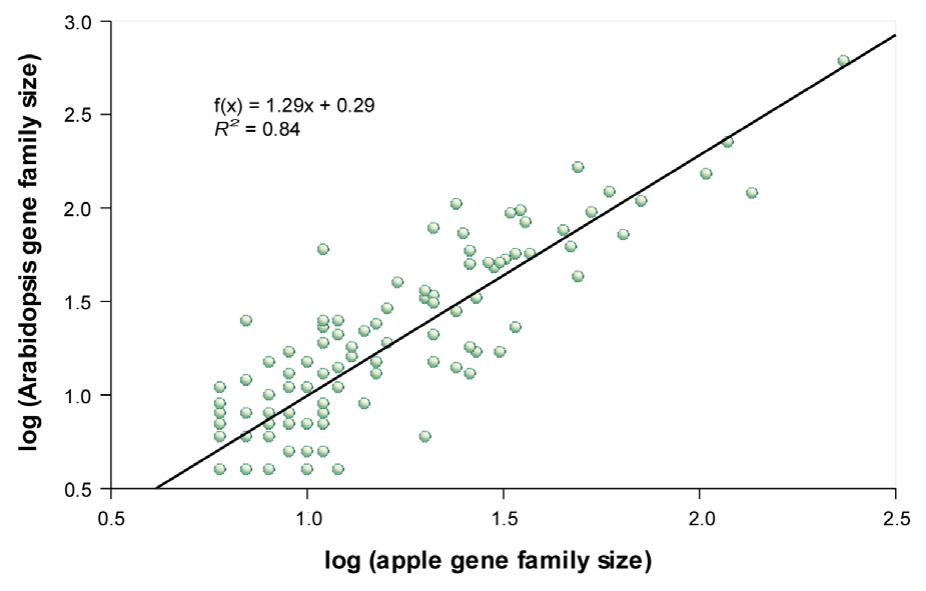
**Comparison of apple and *Arabidopsis *gene family sizes**. Scatter-plot comparing gene family sizes for apple versus *Arabidopsis*. Each point represents a single family. The linear correlation (*r*) and best-fit line are indicated.

The sizes of 111 apple gene families with more than five members (see Additional file 3) were compared with those of *Arabidopsis *(Figure 3). I excluded from this analysis those families characterized as domain- or repeat-containing proteins or as hypothetical proteins. The majority (70 of 111) of the apple families had fewer members than *Arabidopsis*. As the unigene collection only partially represents the gene complement of the apple, this was an expected result; overall apple family sizes should be considered underestimates of the actual sizes for the majority of the families. This deviation is likely particularly acute in families having members with overall low expression or which are expressed in tissues not represented in the EST libraries analyzed in this study. Nevertheless, the gene-copy numbers in the apple and *Arabidopsis *were significantly correlated (Figure 3; *R*^*2 *^= 0.84), fostering two conclusions. First, the overall estimated gene family sizes in the apple seem to remain proportionate with actual sizes. Second, using *Arabidopsis *as a reference, no major changes in gene-copy number seem to have occurred in the apple.

Nevertheless, a number of gene families in the apple were larger than in *Arabidopsis *(Figure 3), suggesting putative amplifications of gene-copy number. Some of these families also had more members than poplar (Table 5; see Additional file 4, 5, 6, 7, 8, 9, 10, 11, 12, 13, 14, 15, 16, 17, 18, 19 and 20), which represents the closest outgroup to the apple with a complete genome sequence, reinforcing the possibility of some gene families being amplified in this species. Monitoring the number of gene duplications in whole families and subsets of young paralogs independently, indicated that these gene families had amplified their members after the origin of the Pyrinae by a ratio ranging from 1.5 and 6 (Table 5), and on average 2.36 folds; suggesting that the amplification of these families likely represents amplifications specific to the Pyrinae/apple genome.

**Table 5:** Amplified apple gene families

Gene family	Apple	*Arab.*	Poplar	Gene-copy number with *K*s < 0.4	Duplicates in young paralogs	Gene family amplification ratio
20S proteasome alpha/beta subunits	29	23	21	18	11	1.61
Chlorophyll a/b-binding proteins	27	21	23	13	14	2.08
β-amylases	18	9	10	10	8	1.80
Elongation factor-1	18	5	13	10	8	1.80
Malate dehydrogenases	14	12	10	8	6	1.75
Glyceraldehyde 3-phosphate dehydrogenase	13	10	10	7	6	1.86
*α*-tubulins	11	8	9	4	7	2.75
S-adenosylmethionine synthetases	10	4	6	6	4	1.67
Vacuolar sorting receptors	8	7	5	3	5	2.67
26S proteasome regulatory subunit (RPN8)	5	2	2	2	3	2.50
Starch phosphorylases	6	2	4	3	3	2.00
40S ribosomal protein S5	6	2	2	1	5	6.00
40S ribosomal protein S4	6	3	5	3	3	2.00
60S ribosomal protein L12	6	3	3	4	2	1.50
ADP-glucose pyrophosphorylase family	5	3	2	3	2	1.67
Aconitate hydratases	5	4	4	2	3	2.50
GDP-mannose 3,5-epimerase	4	1	2	1	3	4.00

Comparative analysis of gene-copy number between apple, *Arabidopsis*, and poplar families. The table includes apple gene families with more members in apple than in *Arabidopsis *and poplar.

Interestingly, some of the amplified families developed related functions, suggesting the amplification of interrelated gene families (see also next section). For example, six families were involved in protein metabolism; these included elongation factor 1 (*eEF1*) and three families encoding ribosomal proteins (*S4*, *S5 *and *L12*), which are all directly involved in protein biosynthesis. Two other families encoded proteasome subunits: the proteasome alpha/beta subunit and the 26S proteasome regulatory subunit, which are involved in controlled proteolytic activity. Of note, two important families encoded proteins involved in the degradation of starch: starch phosphorylase and β-amylase. On the other hand, other families seemed to exhibit a variety of different unrelated functions including diverse metabolic functions or photosynthesis (chlorophyll a/b-binding protein).

Together, these results support the hypothesis that selection acting on duplicated genes resulted in amplification of specific gene families. The proportions of young paralogs within these families were consistent with amplifications most likely specific to the Pyrinae/apple genome.

### Functional bias in the retention of duplicated genes

**Figure 4:**
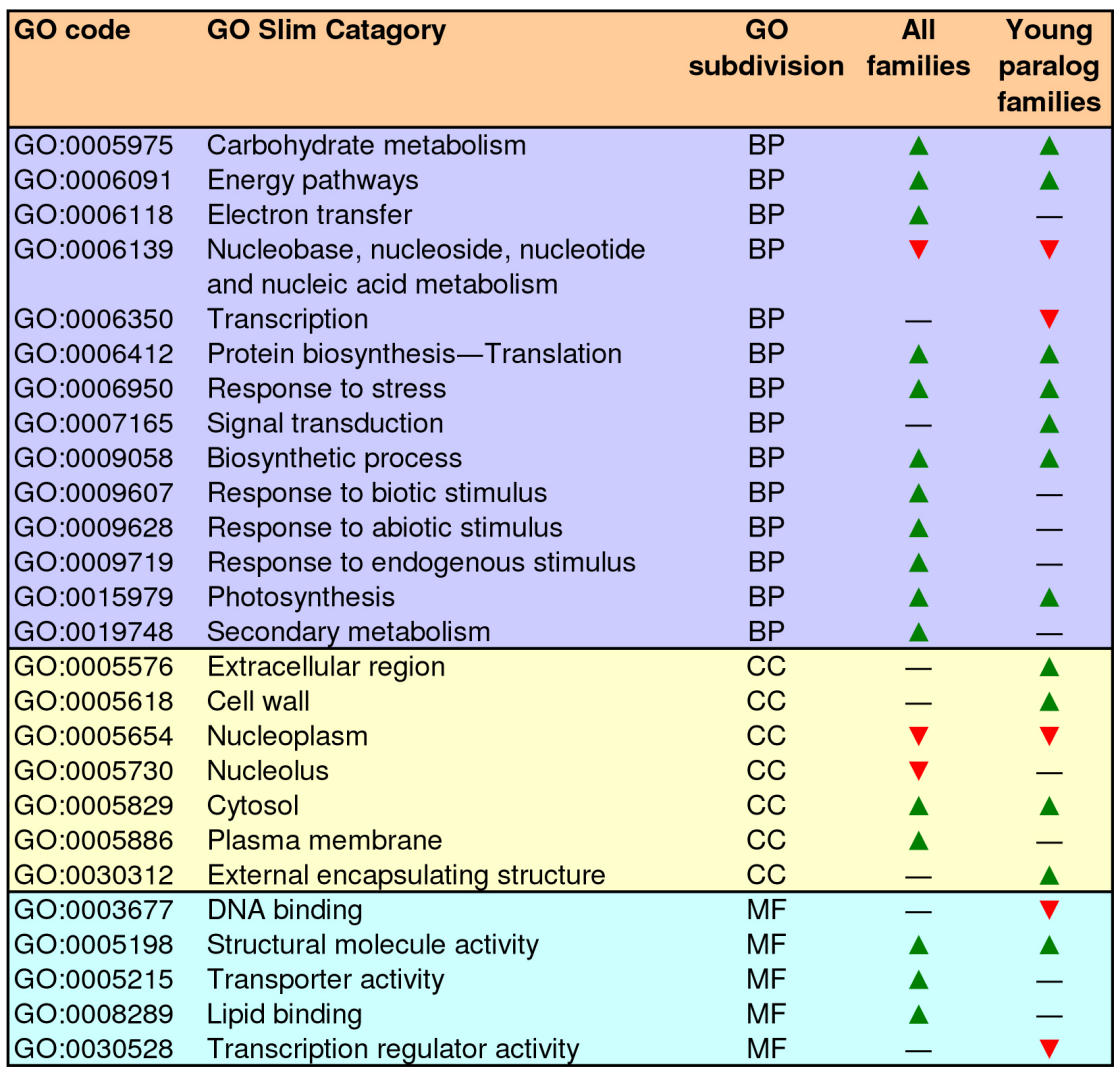
**Functional annotation of apple genes and gene families**. GO categories over-represented (green arrow heads) or under-represented (red arrow heads) in apple gene families as compared with single-copy genes and in young paralog families as compared with single-copy genes and gene families.

The impact of selection on gene duplication can also be interpreted in a broad sense by asking whether the patterns of gene retention/loss have affected entire biological processes, which most often involve evolutionarily unrelated genes and gene families. To examine this possibility, I first identified Gene Ontology (GO) categories that were over-represented or under-represented in gene families as compared with single-copy genes. Annotation of gene families and single-copy genes to the plant GOslim categorization system was performed using Blast2GO and showed that 70% (2998 of 4259) of single-copy genes and 72% (1472 of 2027) of gene families could be associated with at least one GO category. On average, singlets were associated with 3.5 GO terms, whereas gene families were associated with 4.0 GO terms, and the two groups were distributed equally across the three general sections provided by the GO annotation scheme: biological processes, molecular functions, and cellular components (data not shown). Several GO categories were over-represented or under-represented in gene families, which suggested functional bias in the process of gene retention in the apple (Figure 4). Over-represented terms suggested the amplification of genes responsible for certain biological processes that may have been important for adaptation to the medium. Thus, terms like response to biotic and abiotic stimuli and response to stress were all over-represented in gene families. Also, a variety of terms related to metabolic processes, including carbohydrate metabolism, protein metabolism, or secondary metabolism, were over-represented in gene families. Other significantly enriched terms in gene families were those involved to the production of energy, i.e., generation of precursor metabolites or photosynthesis. By contrast, genes related to nucleic acid metabolism seem to have been preferentially lost (Figure 4).

To explore the extent to which this pattern of gene retention might reflect characteristics specific to the Pyrinae/apple genome, over-represented or under-represented GO terms were searched within the group of young paralog families (Figure 4). Some terms that were over-represented in the whole dataset were not in young paralog families (Figure 4); conversely, signal transduction and transcription were over-represented and under-represented, respectively, only in young paralog families (Figure 4). These results highlighted differences in gene retention during or after the formation of the Pyrinae genome. Overall, the biological processes protein biosynthesis, carbohydrate metabolism, energy pathways, photosynthesis, response to stress, and signal transduction were all over-represented among young paralogs (Figure 4). Most remarkably, clear coincidences were observed between these GO terms and the functions of some gene families that underwent recent amplification (Table 5), suggesting that the latter process may be part of a broader network of selective gene retention involving complete biological processes. These coincidences were best illustrated by the large number of amplified gene families involved either in protein biosynthesis (i.e., *eEF1 *and *S4*, *S5*, and *L12 *ribosomal subunits) or carbohydrate metabolism (i.e., *β*-amylase, malate dehydrogenase, Glyceraldehyde 3-phosphate dehydrogenase, starch phosphorylase, ADP-glucose pyrophosphorylase, and GDP-mannose 3,5-epimerase). The *Lhc *family of chlorophyll a/b-binding proteins also constitutes an important photosynthetic gene family. Other families could not be related to any of the biological processes over-represented among young paralogs and may represent cases of selective gene retention only associated with specific families.

It is now well supported that different modes of gene duplication result in the retention of different functional-types of genes [52]. The balanced gene drive model predicts a reciprocal relationship between the genes that are preferentially retained following small-scale duplications and those genes that are preferentially retained following polyploidy [52,53]. For instance, the so called connected genes, which are often dosage-sensitive, are duplicated through polyploidy [18,54]. Duplication through polyploidy allows concerted duplication of interrelated genes and consequently, maintenance of proper gene balance [53]. Genes associated with GO terms like signal transduction, transcription regulation or protein metabolism (i.e. ribosomal proteins or proteasome pathways), are preferentially over-retained postpolyploidy whereas they are under-retained following small-scale duplications (see Freeling [52] for a review). It is likely therefore that genes included in the GO terms signal transduction and protein biosynthesis, which were over-represented among apple young families (Figure 4) may have preferentially duplicated through the action of polyploidy rather than small-scale mechanisms. On the contrary, other genes are prone to duplicate through small-scale events. Well characterized gene families in this group are stress-responsive genes [18,54,55]. Thus, apple genes within the over-represented GO term response to stress (Figure 4), may have preferentially amplified through small-scale events i.e. tandem duplications or transpositions. Gene duplication however, can exhibit complex relationships and it can not be excluded that genes in some families have duplicated through both polyploidy and small-scale mechanisms [52].

### Patterns of gene duplication within gene families

According to the preceding analysis, different mechanisms of gene duplication seemed to have operated in the apple genome, which agrees with the analysis of mixture densities in suggesting that both polyploidy and small-scale duplications may have had a prominent role in gene duplication; particularly after the origin of the Pyrinae lineage (Figure 1). Although the analysis of EST data does not allow conclusive determination of the mechanism by which a gene duplication originated, I inspected gene phylogenies to find clues and make inferences on different modes of gene duplication based on the structure and time of paralog diversification. This section reports such an analysis of three representative gene families.

#### Lhc gene family

Light-harvesting complexes (LHC) of photosystem I (PSI) and II (PSII) contain a group of chlorophyll a/b binding proteins encoded by the *Lhc *gene family. Ten major types of highly abundant Lhc proteins have been identified: four associated with PSI (Lhca1 to 4) and six mainly associated with PSII (Lhcb1 to 6), although Lhcb1 and Lhcb2 can also be involved in PSI. Four additional forms (Lhca5, Lhca6, Lhcb4.3, and Lhcb7) are encoded by genes expressed at very low levels, and their products are still poorly characterized [56]. Twenty-one genes in *Arabidopsis *and 23 genes in poplar encode *Lhc *family members [56].

**Figure 5:**
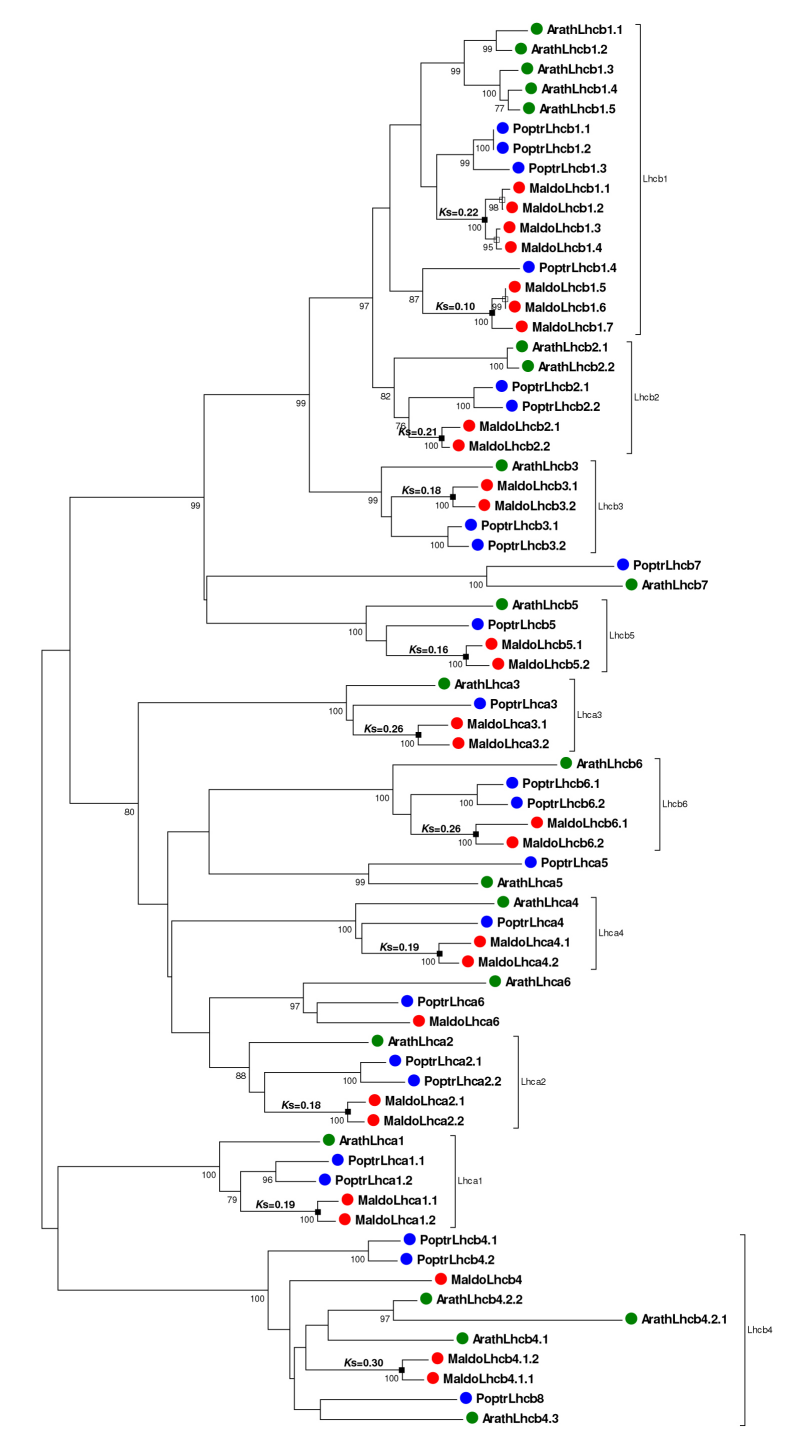
**Phylogenetic analysis of the *Lhc *gene family**. Unrooted neighbor-joining tree generated using the aligned nucleotide sequences of 21 *Arabidopsis *(green dots), 23 poplar (blue dots), and 27 apple (red dots) members of the *Lhc *gene family. Bootstrap values higher than 75 are indicated under each branch. *K*_s _distances between apple paralogs are indicated on the left of each node. Apple gene duplications tentatively assigned to the Pyrinae polyploidization (black squares) or small-scale events (white squares) are marked.

In the apple, the characterization of the *Lhc *family suggested the existence of at least 27 members. The gene genealogy, including the genes from the tree species (*Arabidopsis*, poplar, and apple), suggested that the apple dataset contained more than one member for the 10 major types of Lhc proteins, whereas for the four *Lhc *genes expressed at low levels, only a single ortholog, that of Lhca6, was identified (Figure 5). Absence of characterization of apple orthologs to *Lhca5*, *Lhcb4.3*, and *Lhcb7 *was likely a consequence of their low expression rather than their absence from the apple genome, and therefore the actual number of *Lhc *genes in the apple is likely >27.

A striking feature of the pattern of gene duplications observed for the 10 major *Lhc *genes in apple was that they all exhibited at least one episode of gene duplication after the split of the apple and poplar lineages (Figure 5). To obtain a sense of the time of the *Lhc *gene duplications in apple, synonymous substitutions rates were surveyed individually for each pair of paralogs. The estimated *K*_s _distances (Figure 5) suggested gene duplications of similar ages for the 10 major *Lhc *genes (*K*_s _= 0.16-0.30), which are contemporaries to the Pyrinae episode of polyploidy (Figure 1). This synchrony of parallel gene duplications likely resulted from polyploidy and gene retention for the 10 major groups in the family. The alternative possibility that would involve multiple single-gene duplications occurring at similar times in all members of the family seems improbable.

Polyploidy alone did not seem to explain the entire pattern of gene duplication observed in the *Lhc *family, however. In particular, *Lhcb1 *appears to have been particularly prone to the accumulation of recent gene duplications, a trend that seems to have also occurred in *Arabidopsis *and poplar (Figure 5). Therefore, the occurrence of small-scale duplications in addition to polyploidy likely explains the pattern of gene duplication within the *Lhcb1 *clade (Figure 5).

#### Tubulin subfamily

In contrast to the *Lhc *family, the apple tubulin phylogeny suggested a distinct mode of gene duplication that seemed to be consistent with different patterns of gene duplication occurring in different parts of the family. Tubulins, which are the structural components of microtubules, are organized in heterodimers containing the two major tubulin forms: α and β. *Arabidopsis *and poplar tubulin families consist of different gene-copy numbers of the two subfamilies: six α-tubulins and eight β-tubulins in *Arabidopsis*, and nine α-tubulins and 20 β-tubulins in poplar [57].

**Figure 6:**
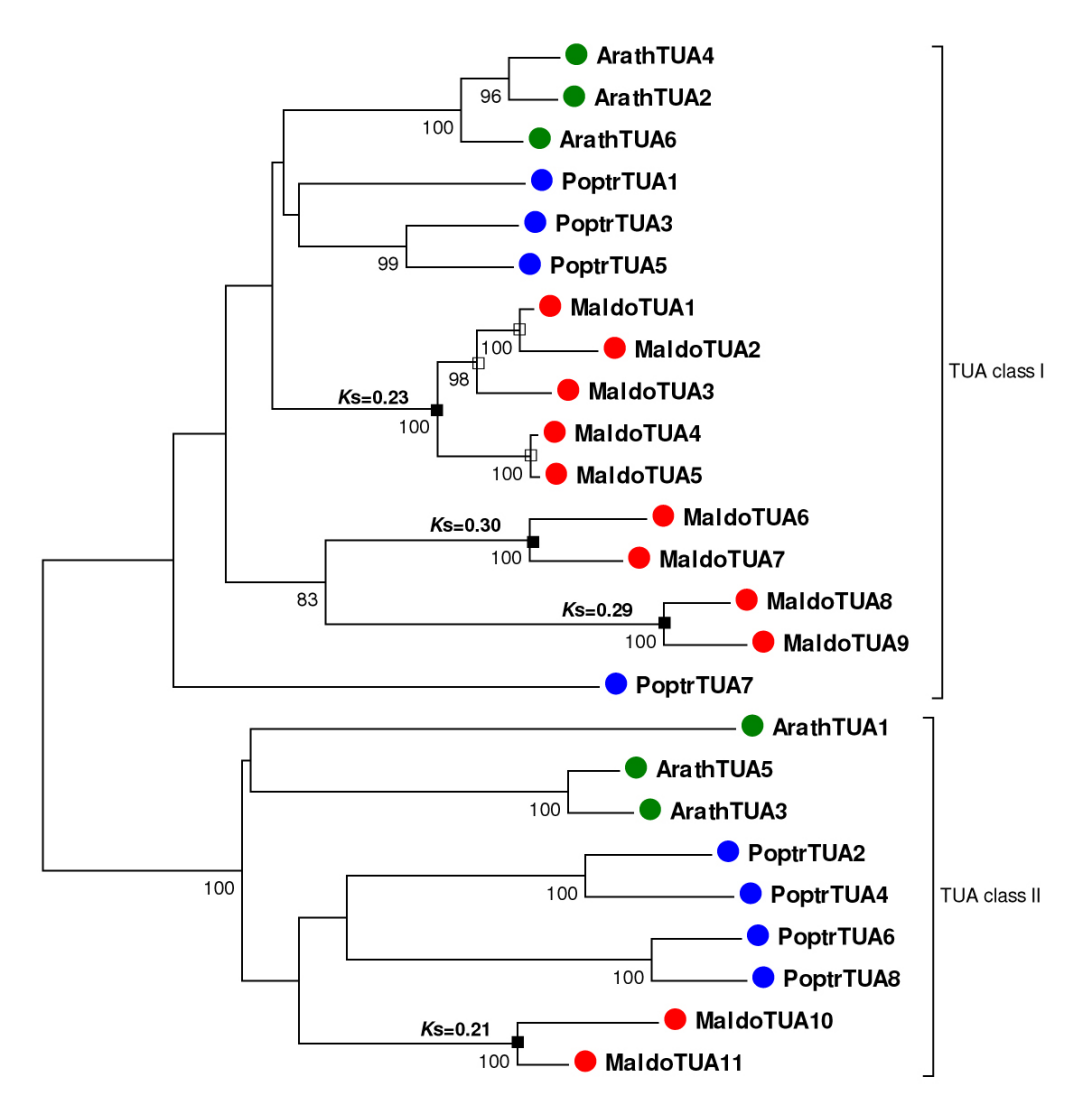
**Phylogenetic analysis of the *α*-tubulin gene subfamily**. Unrooted neighbor-joining tree generated using the aligned nucleotide sequences of 6 *Arabidopsis *(green dots), 9 poplar (blue dots), and 11 apple (red dots) members of the *α*-tubulin gene subfamily. Bootstrap values higher than 75 are indicated under each branch. *K*_s _distances between apple paralogs are indicated on the left of each node. Apple gene duplications tentatively assigned to the Pyrinae polyploidization (black squares) or small-scale events (white squares) are marked.

**Figure 7:**
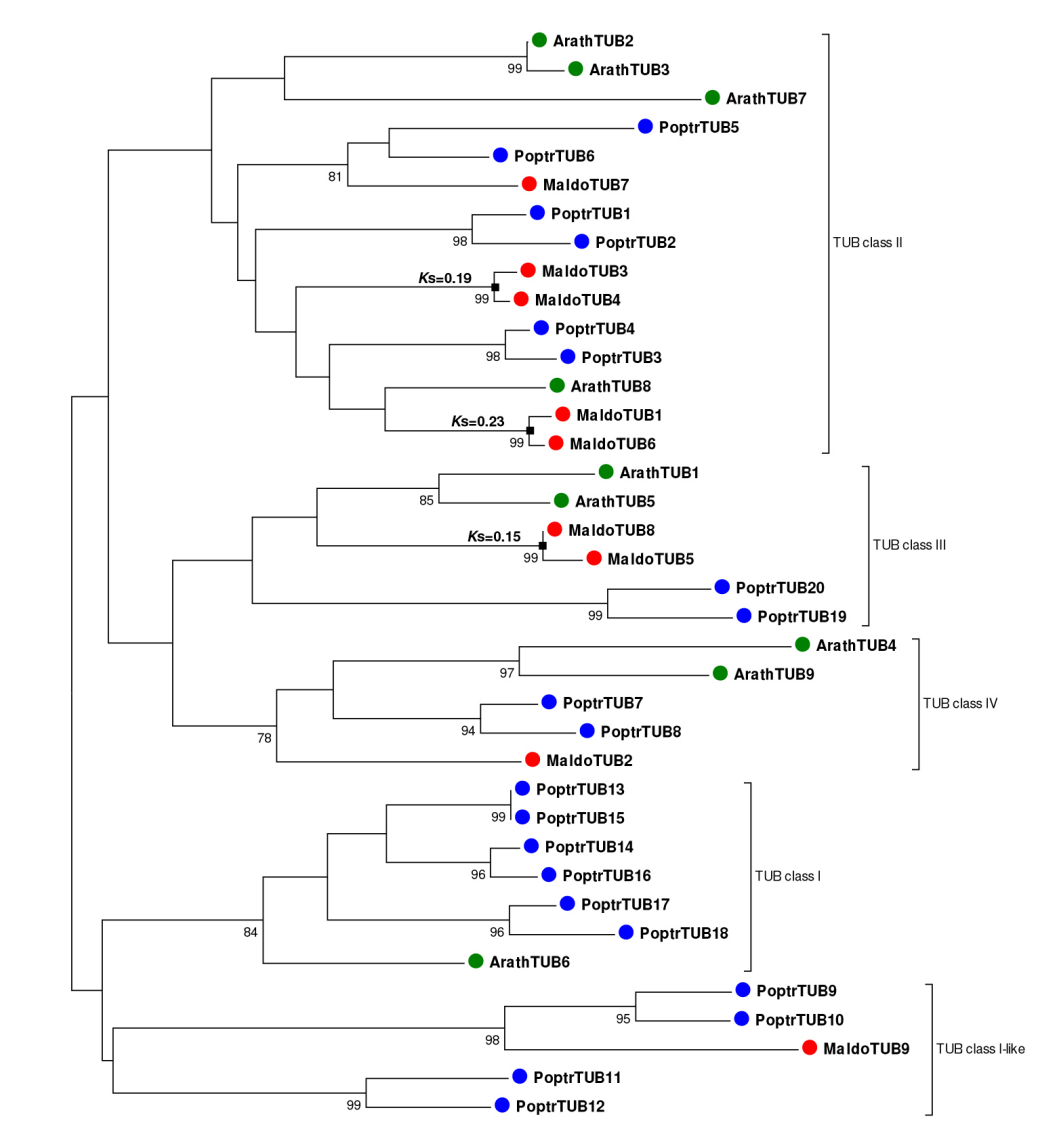
**Phylogenetic analysis of the *β*-tubulin gene subfamily**. Unrooted neighbor-joining tree generated using the aligned nucleotide sequences of 8 *Arabidopsis *(green dots), 20 poplar (blue dots), and 9 apple (red dots) members of the *β*-tubulin gene subfamily. Bootstrap values higher than 75 are indicated under each branch. *K*_s _distances between apple paralogs are indicated on the left of each node. Apple gene duplications tentatively assigned to the Pyrinae polyploidization (black squares) or small-scale events (white squares) are marked.

In the apple, characterization of the tubulin gene family identified 11 α-tubulins and 9 β-tubulins, suggesting amplification in gene-copy number only for the *α*-tubulins. Phylogenetic analysis, including genes from the three species (Figure 6), divided the α-tubulins into two known classes, I and II [57]. α-tubulin members in both *Arabidopsis *and poplar were represented equally among the two classes (Figure 6); in apple however nine α-tubulins were clustered within class I, whereas only two members were clustered within class II (Figure 6). This bias in the distribution of *α*-tubulins seemed to be the result of the differential amplification of the class I members. *K*_s _times assigned to the nodes linking apple paralogs indicated that, similar to the *Lhc *family (Figure 6), the pattern of gene duplications observed for the α-tubulins could be explained if paralogs were derived both from the Pyrinae polyploidization and from small-scale duplications (Figure 6). By contrast, reconstruction of the β-tubulin phylogeny (Figure 7) suggested a different scenario with fewer events of recent gene duplications. Hypothetically, from the six putative β-tubulins present before the Pyrinae polyploidization, only three of them may have retained a paralog, and gene loss was likely the fate of the other three (Figure 7).

Interestingly, the pattern of gene duplication observed for the apple tubulins in which only α-tubulins have undergone amplification is virtually the inverse situation that led to the differential expansion of the β-tubulin subfamily in poplar [57].

#### 40S ribosomal protein S5

The *S5 *ribosomal gene family encodes a component of the 40S subunit of cytosolic plant ribosomes [58]. Characterization of the *S5 *ribosomal gene family identified at least six members in apple, a gene-copy number that substantially outperforms the size of the *Arabidopsis *and poplar families, each of which contains only two members.

**Figure 8:**
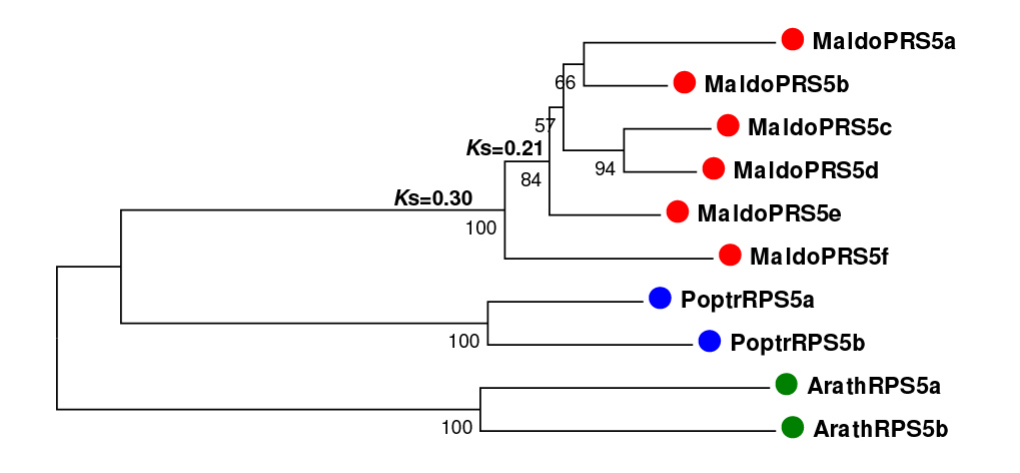
**Phylogenetic analysis of the ribosomal *S5 *gene family**. Unrooted neighbor-joining tree generated using the aligned nucleotide sequences of 2 *Arabidopsis *(green dots), 2 poplar (blue dots), and 6 apple (red dots) members of the ribosomal *S5 *gene family. Bootstrap values higher than 75 are indicated under each branch. *K*_s _distances between apple paralogs are indicated on the left of each node. Apple gene duplications tentatively assigned to the Pyrinae polyploidization (black squares) or small-scale events (white squares) are marked.

The gene genealogy, including the *S5 *genes from the three species, grouped the six apple paralogs into a single clade with coalescence *K*_s _time of 0.3 (Figure 8). Moreover, all the internal nodes exhibited *K*_s _times ranging from 0.12 to 0.2. The short time in which this gene family seems to have accumulated a large number of gene duplications makes it difficult to predict which of them, if any, was related to the Pyrinae polyploidization. Nevertheless, if it is assumed that one duplication was the result of polyploidy, three or four additional single-gene duplications should be considered to explain the pattern of diversification of the apple *S5 *ribosomal gene family (Figure 8).

Collectively, these results suggested that the patterns of gene duplication observed in apple were the result of the combined action of both polyploidy and small-scale duplications. The pattern observed for the *Lhc *family showed how polyploidy may have played a role in the simultaneous amplification of the 10 major members of the family. Like many other nuclear encoded genes that function in organellar macromolecular complexes, *Lhc *genes may be dosage-sensitive and members in the family might have been retained collectively following the most recent WGD, to keep proper protein balance [53]. In contrast, the mode of diversification observed for the *S5 *ribosomal component emphasized the role that the recurrent action of small-scale duplications have had on the amplification of this family. Ribosomal proteins seem to exhibit different patterns of duplication among species. While in *Arabidopsis *ribosomal proteins were collectively over-retained after the most recent polyploidization in the species, poplar or rice genomes do not show evidence of over-retention of ribosomal proteins after WGD [52]. Interestingly, the recent analysis of ribosomal paralogs in *Brassica napus *indicated that some ribosomal genes have amplified through small-scale events rather than polyploidy [59], suggesting a parallelism with the pattern of duplication of the apple *S5 *protein. Finally, the interplay of polyploidy, small-scale duplications, and the different modes of gene retention/loss led to the distinct profiles exhibited by different groups of tubulins.

### Gene expression divergence between paralogs

Gene duplication generates genetic redundancy, thereby allowing functional diversification. The evolution of duplicated genes by developing divergent patterns of gene expression has been suggested to be a first step in this process, allowing the partition of gene function (subfunctionalization), thus favouring gene retention and ultimately creating the conditions for neofunctionalization [60,61]. By comparing the expression profiles of 1648 pairs of paralogs in 14 libraries (see Additional file 2) representing different tissues and/or developmental stages, I assessed the extent to which duplicated genes in the apple have developed divergent patterns of gene expression.

**Figure 9:**
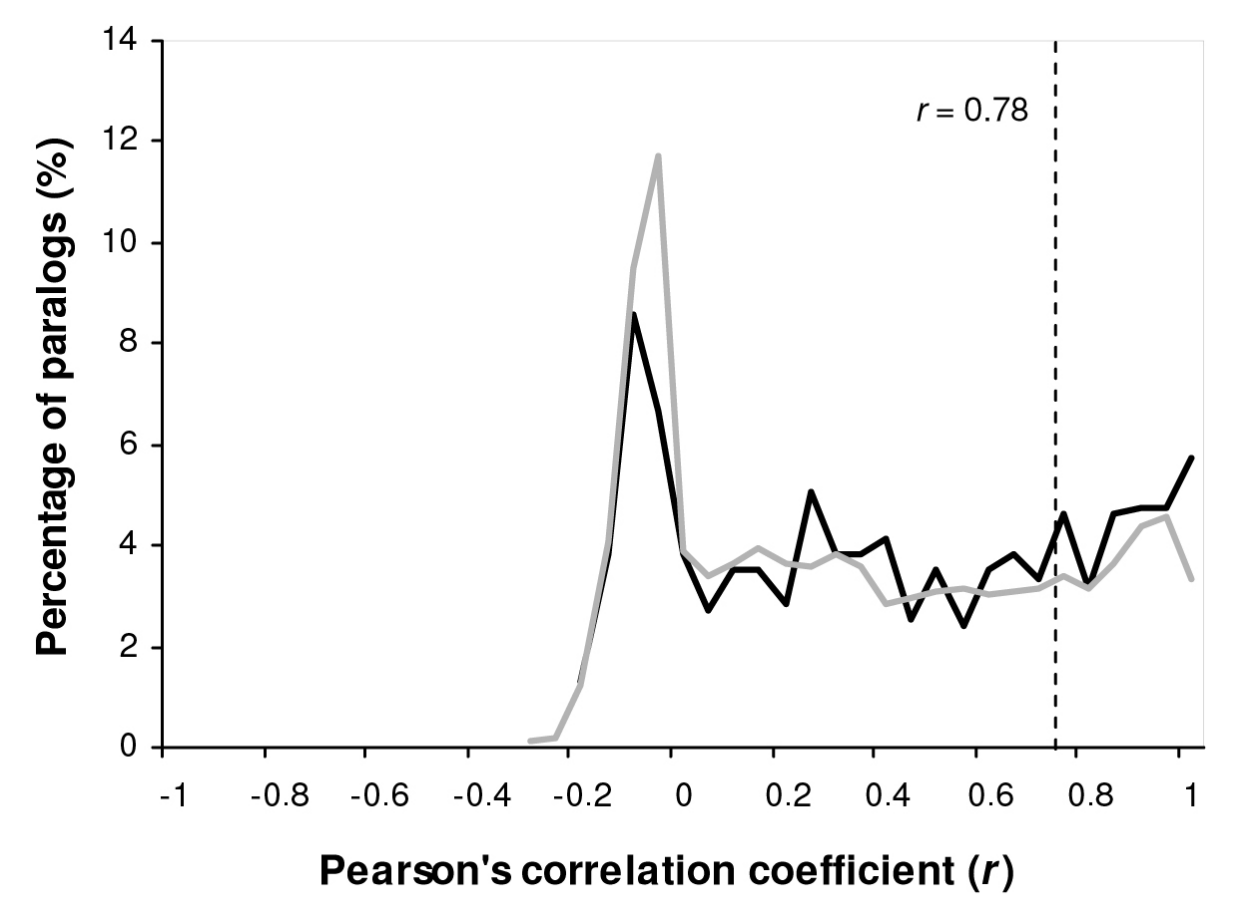
**Gene expression divergence between apple paralogs**. Frequency distributions of the Pearson's correlation coefficients computed with the expression profiles of pairs of duplicated genes. Paralogs showing *K*_s _≤ 0.4 (gray line) and *K*_s _> 0.4 (black line) are shown. The dotted vertical line (*r *= 0.78) represents the threshold value used to consider two genes as being significantly co-regulated (*α *= 0.05).

Pairs of duplicated genes showed Pearson's correlation coefficients ranging from -0.33 to +1, of which 82% exhibited values lower than the cutoff (*r *= 0.78) estimated by considering two genes as being significantly co-regulated (α = 0.05). This proportion of genes showing divergent patterns of gene expression seemed to be consistent with estimates made for other species with proportions usually ranging from 74% to 85% [13,20,61,62]. Divergence in expression between duplicated genes was further analyzed separately for the subset of young paralogs (*K*_s _< 0.4) and their older counterparts (Figure 9). Interestingly, there were only slight apparent differences between the two datasets, as the frequency distributions of both groups exhibited similar shapes (Figure 9) and the proportions of genes showing significantly divergent expression profiles were very close: 80% for young paralogs and 83% for older paralogs. These results suggested similar patterns of divergence in gene expression regardless of the age of the gene duplication. This possibility was confirmed by the absence of a correlation between the Pearson's *r *values of gene expression and the evolutionary divergence (*K*_s_) between paralogs (*r *= -0.05; see Additional file 21).

A closer look at the frequency distributions of the Pearson's correlation coefficients suggested that those pairs showing *r *values ranging from 0.2 to 1 were equally distributed, whereas a larger proportion of paralogs showed correlation values close to zero (Figure 9); evidenced by sharp peaks in the distributions from -0.2 <*r *< 0.1. Both groups of paralogs showed this pattern although it was more pronounced for young paralogs (Figure 9). This profile may indicate major changes in gene expression between paralogs by developing shifts towards tissue-specific expression, which is a characteristic of subfunctionalization transitions. To further evaluate this possibility, the 14 EST collections were further classified according to four tissue types (fruit, vegetative, reproductive, and vascular), and the tissue-specific expression of the 631 pairs of young paralogs was evaluated using Fisher's exact test. For 25% of the gene pairs analyzed, a significant change towards tissue-specific expression or suppression was observed.

**Figure 10:**
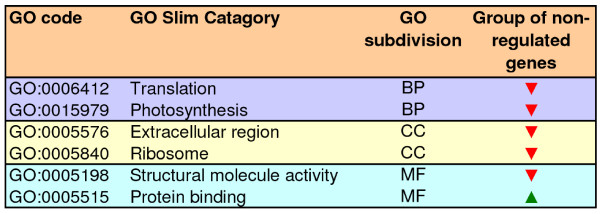
**Functional annotation of apple paralogs with divergent expression profiles**. GO terms over-represented (green arrow heads) or under-represented (red arrow heads) in apple paralogs exhibiting divergent patterns of gene expression.

Finally, I studied whether gene expression divergence was affected by the gene function of paralogs. For this, an enrichment analysis of GO terms was performed between, the group of paralog genes showing divergent patterns of gene expression and the group of duplicates showing co-regulated expression profiles. The results from this analysis are presented in figure 10. Overall, the vast majority of the GO categories were equally distributed among the two groups of paralogs. Still, the biological processes translation and photosynthesis were significantly under-represented within the group of paralogs exhibiting divergent expression profiles (Figure 10).

## Conclusions

Here I have studied the mode of gene duplication in the apple by analyzing EST data. Characterization of the age of gene duplications allowed identification of the major events that have contributed to the formation of duplicated genes in the species, namely a continuous mode of small-scale duplications and two episodes of large-scale duplications likely corresponding to two independent polyploidizations. I studied the signatures that this pattern of gene duplication has left in the apple genome, with a special emphasis on genes duplicated during or after the formation of the Pyrinae lineage. The results obtained suggested that the process of gene duplication has shaped the apple genome in different ways, including the selective retention of paralogs associated with specific biological processes, the amplification of gene families, and mediating extensive subfunctionalization between paralogs. The mode of gene duplication showed that both polyploidy and small-scale duplications are responsible for the comparatively high number of duplicated genes that have originated since the formation of the polyploid ancestor of the Pyrinae. Thus, the phylogenies of the gene families analyzed were best explained by a combination of both processes. The completion in the near future of the apple genome sequence [63] will allow better evaluation of this possibility and will show more details of the mechanisms and timing of gene duplication in the species.

The observed bias in the retention of duplicated genes suggested that selection favoured specific biological processes and/or gene functions and raised the question of the biological meaning of gene duplication, not only for the evolution of the apple but also for diversification within the context of the Pyrinae group, which has a common polyploidy origin. Thus, for example, it is intriguing that a number of metabolic processes (i.e., carbohydrate metabolism, protein metabolism, and photosynthesis) were all over-represented among young paralogs, a pattern of gene retention that seems to have had a role in the amplification of a number of gene families. The origin of the Pyrinae lineage through polyploidy may be somewhat related to this observation. Although the mechanisms by which polyploids develop phenotypic novelty are still largely unknown, it is well accepted that increased growth vigor is seen in polyploids, which is apparently a key aspect of success under natural conditions [64]. A recent report by Ni et al. [65] showed that increased vigor and biomass production in resynthesized allopolyploids of *Arabidopsis thaliana *and *Arabidopsis arenosa *are mediated by upregulation of genes involved in photosynthetic and carbohydrate metabolic pathways that led these polyploids to produce more chlorophyll and accumulate more starch than their parents [65]. Interestingly, the observation that duplicated apple genes related to photosynthesis and carbohydrate metabolism, may have been preferentially retained during the polyploidy formation of the apple genome suggests a parallelism with these findings. The possibility that this pattern of paralog retention may have increased the fitness of the newly formed Pyrinae polyploid ancestor by modifying its photosynthetic and metabolic machineries is a hypothesis that may provide a connection between the general belief of adaptive superiority of polyploids with the evolutionary success of a plant lineage that originated via allopolyploidization.

An alternative hypothesis may also relate the pattern of gene retention observed in the apple with the acclimatization to a seasonal habitat because extant temperate deciduous Pyrinae species likely descended from Tertiary tropical or subtropical ancestors. Fossil records date the origin of Pyrinae to the middle Eocene [36]. At that time, the Earth was dominated by a humid tropical climate [66], and members of the Pyrinae were primarily distributed in the northern hemisphere and were particularly well represented among the boreotropical floras of eastern Asia and North America [67]. During the late Eocene-Oligocene transition, global cooling caused the disintegration of the boreotropical flora and the retreat of many taxa towards refugees near the equator [68]. The presence of the extant genera *Eriobotrya *and *Rhaphiolepis *in the tropical forests of China, Indochina, and Malaysia illustrate this process within the subtribe Pyrinae [67]. For the ancestors of other species of the subtribe like the apple that became prominent members of modern temperate deciduous forests, adaptation to a seasonal environment with cooler average temperatures and cold winters was likely an important aspect of success. Interestingly, extensive experimental evidence is accumulating that reinforces the notion that metabolic changes, including those of proteins, carbohydrates, and photosynthesis, are central to seasonal adaptability and cold acclimatization. For example, the role of carbohydrate metabolism in the acquisition of cold hardiness [69-72] and in the adaptation of deciduous trees to meet the seasonal changes in energy requirements [73] is well known. In addition, protein metabolism has been related to the high protein turnover exhibited by deciduous trees across different seasons [12]. Furthermore, the distinct regulation of photosynthesis in response to temperature has been suggested to delineate the differences in the potential for cold acclimatization between temperate and tropical species [74]. Such an scenario would agree with the recent proposal by Van de Peers and co-workers which have suggested that polyploids could greatly enhance the diversification potential of those lineages that have succeed during periods of environmental instability [75,76].

Another remarkable feature of gene duplication in the apple was the high proportion of paralogs showing divergent patterns of expression, in some cases by developing bias towards tissue-specific expression/suppression. This behaviour was found to be similar regardless of the age of paralog formation, which suggested evolution by subfunctionalization early after gene duplication. Extensive subfunctionalization may have set the basis for gene diversification and the development of novel gene functions. These might have been mechanisms that played a role in the acquisition of new traits specific to the apple or the Pyrinae lineage and thus merit further research. Recent experimental evidence has suggested that phenotypic novelty can evolve quickly through changes in the pattern of gene expression of duplicated genes [64,77].

With the advent of more information about the biology of model organisms, researchers are using comparative genomic approaches to make functional knowledge portable across species. Comparative genomics largely relies on the characterization of orthologs-those genes derived from a speciation event that are therefore likely candidates to develop conserved functions [78]. The dynamic nature of plant genomes in terms of gene duplication, retention, and loss of paralogs and subsequent diversification is, however, challenging this view and suggests new questions into the functional equivalence of orthologous genes [61]. The apple has emerged as the model system for the Pyrinae subtribe [63], and as a consequence research on other species of the subtribe will take advantage of the resources and information developed for the apple. The rich population of duplicated genes that seems to have diversified in the apple genome, particularly after the formation of the Pyrinae lineage, questions the extent to which this pattern of gene duplication is shared by other members of the subtribe or whether gene duplication has proceeded in different ways in different species, acting as the substrate for genetic and phenotypic diversification. These are questions that should be considered when undertaking comparative genomic studies between species belonging to this taxonomic group.

This study provides for the first time a genome-wide characterization of the mode of gene duplication in the apple, a major fruit crop and representative of the subtribe Pyrinae—important taxonomic group of the Rosaceae family.

## Methods

### Construction of unigenes

EST sequences from 33 non-normalized libraries obtained from different tissues (see Additional file 2) from the apple cultivar Royal Gala were downloaded from GenBank. For unigene construction, all libraries were merged into a single dataset of 119,177 ESTs. Low-quality sequences or those shorter than 100 nt were removed using Seqclean [79]. The resulting EST collection was clustered and assembled using the TGICL program under stringent conditions (clustering options were set to group sequences with at least 98% identity in at least 40 nucleotides with no more than 20 nucleotides from the sequence end) to minimize clustering of close paralogs as much as possible [79,80]. After assembly, the resulting dataset consisted of 33,211 unique sequences (unigenes) containing 13,168 contigs (derived from clusters of more than one EST) and 20,043 singletons (solitary ESTs). Unigenes corresponding to putative protein-coding genes were identified using stand-alone BLASTX v. 2.2 searches against the plantRefSeq protein database with default settings and an E-value cutoff of 1e-10 [81]. Only those sequences aligned with their best match over a length of at least 100 amino acids and showing a protein sequence identity >30% were selected for further analysis.

### Identification and dating of gene duplications

Unigenes corresponding to pairs of duplicated genes were characterized according to the approach reported by Blanc and Wolfe [25]. The open reading frame for each unigene sequence was deduced with GeneWise2.2.0 [82] using its corresponding best-match protein in the plantRefSeq database (NCBI) as a guide. The highest scoring GeneWise DNA-protein alignment was used to obtain: (1) the unigene nucleotide protein-coding sequence, and (2) its translated amino acid sequence after removing N and frameshift-sites containing codons. The collection of protein sequences thus obtained was used for all-against-all sequence similarity searches using stand-alone BLASTP v. 2.2 with default settings and an E-value cutoff of 1e-10 [81]. Pairs of sequences aligned over a length of at least 100 amino acids and showing a sequence identity >30% were considered to be paralogous genes. Of these, only those pairs showing protein sequence identities of at least 60% were used for *K*_s _estimation.

For each pair of paralogs, their deduced protein sequences were aligned using ClustalW [83], and the corresponding nucleotide sequences were aligned accordingly. After removing gap positions, the rate of substitutions per synonymous site (*K*_s _divergence) between each pair of sequences was estimated using the maximum likelihood method of Goldman and Yang [84] as implemented in the codeml program of the PAML package [85] under the F3 × 4 model. *K*_s _values >2 were discarded from further analysis as they may be associated with uncertainty due to saturation of substitutions [25]. For each pair of sequences, *K*_s _values were computed five times, and the estimation showing the best likelihood score was retained for further analysis.

The dataset was cleaned according to the two criteria used by Blanc and Wolfe [25]. First, all unigenes identified as putatively corresponding to transposable elements during BLAST searches or GO annotation, were removed. Second, for each pair of paralogs showing no synonymous substitutions (*K*_s _= 0), one of the two sequences was discarded from the dataset as they were likely multiple entries of the same gene (i.e., alternatively splice variants). Finally, redundant *K*_s _values corresponding to the same duplication event within a gene family were omitted by computing mean values for each node of the gene family phylogenies. For this, gene families were constructed by using single-linkage clustering, and the gene topology was ordered based on *K*_s _distances between paralogs.

### Mixture density of the age distribution of gene duplications

Gene duplications in a genome can originate from a continuous mode of small-scale duplications and discrete episodes of large-scale duplications (i.e., large segmental duplications, aneuploidy, or polyploidy). As a consequence, the age distribution of gene duplications closely follows a mixture of an exponential distribution representing the constant birth and death process of small-scale duplications and normal distributions representing discrete bursts of duplicated genes derived from large-scale duplication events [27]. To test the best fit of the age distribution of apple gene duplications, mixture densities of one exponential and a variable number (0 to 3) of normal distributions were generated, and the goodness of fit of the simulated densities and the observed apple *K*_s _distribution were evaluated using the Kolmogorov-Smirnov test.

### Characterization of gene families

Gene families were constructed based on protein sequence similarity. For this, pairs of paralogs were grouped using a single-linkage clustering approach. Simple sequence counting data provided initial estimates of gene family sizes. These estimates were directly used to calculate the gene family size distribution. The unigene collection only partially represents the gene complement of the apple. To evaluate the extent to which gene family sizes could be biased due to differences in gene expression, I investigated any relationship between gene expression levels and gene copy-number (see Additional file 22). This analysis did not support a positive relationship between gene family size and gene expression; rather highly expressed genes were more frequently represented among single-copy genes or gene families with fewer members (see Additional file 22). Collectively, these observations suggested that the gene family size estimations were not affected by differences in gene expression.

To evaluate putative events of gene family amplification in the apple, the gene-copy numbers of families with more than five members were compared with the gene family sizes of *Arabidopsis*. Families that were larger in the apple were further compared with those of poplar, the closest outgroup to the apple with a complete genome sequence. Amplified gene families were investigated for patterns of recent expansion. For this, the sets of young paralogs (*K*_s _distances lower than 0.4) were selected from the whole dataset and were newly clustered into gene families following the same approach used when building the families from the whole dataset. Hierarchical reconstruction of gene families was used to estimate the proportion of gene duplications for each gene family in the whole dataset and in the group of young paralogs.

A drawback associated with the analysis of ESTs is that, within a gene family, some unigenes may correspond to partial sequences, making it difficult to construct alignments of sufficient length for phylogenetic reconstruction. Thus, for phylogenetic analysis, unigene sequences were manually assembled de novo using all the apple EST resources available in dbEST. Deduced protein sequences were aligned using ClustalX. Protein sequence alignments were further inspected manually and were used as a reference for the alignment of their corresponding nucleotide sequences (see Additional files 23, 24, 25 and 26). Phylogenetic trees were constructed using three different computational methods; the neighbour-join (NJ) method, Bayesian inference (BA) and maximum parsimony (MP). NJ trees were constructed using complete deletion data and p-distances calculated using the three codon positions as implemented in MEGA4 [86]. BA inference of phylogeny was conducted using MrBayes 3.1 [87] under the GTR model with gamma-distributed rate variation across sites. BA trees were visualized with the TreeView software [88]. Finally, MP trees were constructed with MEGA4 [86], using the close-neighbour-interchange algorithm; initial trees were obtained with the random addition of sequences for 10 replicates. Statistical support of the reliability of the trees was obtained from bootstrap analysis with 1000 replications. The three methods yielded basically the same phylogenies, only showing different topologies at the less supported internal nodes. Most remarkably, clustering of recent paralogs was identical using the three methods. For this reason only NJ trees are represented (Figure 5). The phylogenies constructed using MP and BA inference, are available from the author upon request.

### Functional annotation of genes and gene families

EST-derived unigenes often represent partial gene sequences, thereby limiting gene annotation based only on protein-domain information. In this study, I used a GO annotation procedure based mainly on overall sequence similarity using BLAST searches [81]. Translated unigene sequences were functionally annotated to the GO categories included in PlantGOSlim [89] using Blast2GO [90]. The GO annotation scheme provides structured terms that describe gene products based on their associated biological processes, cellular components, and molecular functions. Blast2GO performs GO annotation through BLAST searches against a database and maps positive hits to GO categories. A mapped GO term is assigned to a gene product if it satisfies a pre-established annotation rule. To complement the annotation procedure based on BLAST, Blast2GO also incorporates an InterPro search functionality to add protein domain information.

A standard strategy for gene annotation was followed in this study as outlined by Götz et al. [91]. Briefly, BLAST searches, GO mapping, and the annotation step were performed using the default parameters, except that the e-value cutoff used to retain BLASTP significant hits was set at 1e-10. Those sequences that failed to be annotated based on BLAST searches were further evaluated for protein domain information extraction in the InterPro database using InterProScan. The results obtained from annotating the sequences using these two independent approaches were merged into a single dataset. Finally, the Annex function was used in an augmentation step based on the pre-established relationships of molecular function terms involved in biological processes or acting in cellular components [92]. Genes assigned to a particular GO category were also included in all parental categories. Gene families were annotated with a GO category if at least 30% of the members in the gene family were annotated to that particular category [93]. To evaluate statistical differences in enrichment of GO categories between two groups of genes and/or gene families, Fisher's exact test as implemented in Blast2GO was applied (*P *< 0.01).

### Digital expression analysis

The expression profile for each unigene was obtained by evaluating its EST representation among 14 datasets derived from different tissues and/or developmental stages (see Additional file 2). For this, the 'ace' file generated by the TGICL program during the clustering and assembly project was parsed to extract EST counting data. Only unigenes represented by at least three EST sequences in the whole dataset were selected for analysis. Co-regulation or divergence in expression between pairs of duplicated genes was assessed by computing the Pearson's correlation coefficient (*r*) [32], which generates values ranging from -1 to 1. Values close to 0 indicate strong divergence in the pattern of gene expression of two paralogs, whereas positive values close to 1 indicate co-regulated patterns of gene expression. Negative values indicate evolution towards inverted patterns of gene expression. The *r *value below which expression divergence could be considered statistically significant was defined according to Blanc and Wolfe [61]. For this, the frequency distribution of *r *values calculated for 10,000 randomly selected gene pairs was constructed. The frequency distribution indicated that 95% of the randomly selected gene pairs had an *r *< 0.78. Thus, duplicated genes with *r *≥ 0.78 were considered to be significantly co-regulated (*α *= 0.05). Conversely, paralogs with *r *< 0.78 were considered to have developed divergent patterns of gene expression.

To evaluate tissue-specific expression, the 14 EST datasets were further classified according to the four tissue types represented (fruit, vegetative, reproductive, and vascular). EST counting data for each unigene were extracted from the four datasets as described before, and Fisher's exact test was applied to a 2 × 2 contingency table where genes were assigned to rows and the tissue type to be tested and the bulk of all the other tissue types were assigned to columns.

Custom Perl scripts and SQL database searches were used for dataset management and protocol automation.

## Supplementary Material

Additional file 1 Unigene collection derived from assembling 119,177 apple ESTs cv. 'Royal Gala'. Click here for file

Additional file 2 'Royal Gala' apple EST libraries used in this study. Click here for file

Additional file 3 Apple gene families with more than five members Click here for file

Additional file 4 Apple 20S proteasome gene family members. Click here for file

Additional file 5 Apple Chlorophyll a/b-binding protein gene family members. Click here for file

Additional file 6 Apple β-amilase gene family members. Click here for file

Additional file 7 Apple Eukariotic elongation factor 1 gene family members. Click here for file

Additional file 8 Apple Malate deshydrogenase gene family members. Click here for file

Additional file 9 Apple Glyceraldehyde 3-phosphate dehydrogenase gene family members. Click here for file

Additional file 10 Apple α-tubulin gene family members. Click here for file

Additional file 11 Apple S-adenosylmethionine synthetase gene family members. Click here for file

Additional file 12 Apple Vacuolar sorting receptor gene family members. Click here for file

Additional file 13 Apple 26S proteasome regulatory subunit (RPN8) gene family members. Click here for file

Additional file 14 Apple Starch phosphorylase gene family members. Click here for file

Additional file 15 Apple 40S ribosomal protein S5 gene family members. Click here for file

Additional file 16 Apple 40S ribosomal protein S4 gene family members. Click here for file

Additional file 17 Apple 60S ribosomal protein L12 gene family members. Click here for file

Additional file 18 Apple ADP-glucose pyrophosphorylase gene family members. Click here for file

Additional file 19 Apple Aconitate hydratase gene family members. Click here for file

Additional file 20 Apple GDP-mannose 3,5-epimerase gene family members. Click here for file

Additional file 21 Scatter-plot of Ks distance (synonymous substitution/synonymous sites) between pairs of duplicated genes, plotted against divergence in gene expression (Pearson's correlation coefficient). Click here for file

Additional file 22 **Scatter-plot representing gene family size plotted against gene expression. (A) Individual values represent mean EST counts for each gene family.** (B) Individual values represent raw EST counts for each unigene within each gene family. Click here for file

Additional file 23 Apple, *Arabidopsis *and poplar aligned nucleotide sequences, used for phylogenetic analysis of the Chlorophyll a/b-binding protein (Lhc) gene family. Click here for file

Additional file 24 Apple, *Arabidopsis *and poplar aligned nucleotide sequences, used for phylogenetic analysis of the α-tubulin gene family. Click here for file

Additional file 25 Apple, *Arabidopsis *and poplar aligned nucleotide sequences, used for phylogenetic analysis of the β-tubulin gene family. Click here for file

Additional file 26 Apple, *Arabidopsis *and poplar aligned nucleotide sequences, used for phylogenetic analysis of the 40S ribosomal protein S5 gene family. Click here for file

## References

[B1] Sémon M Wolfe KH Consequences of genome duplication Curr Opin Genet Dev 2007 17 6 505 12 10.1016/j.gde.2007.09.007 18006297

[B2] Zhang J Evolution by gene duplication: an update Trends Ecol Evol 2003 18 6 292 298 10.1016/S0169-5347(03)00033-8

[B3] Sankoff D Gene and genome duplication Curr Opin Genet Dev 2001 11 6 681 684 10.1016/S0959-437X(00)00253-7 11682313

[B4] Hurles M Gene duplication: The genomic trade in spare parts PLoS Biol 2004 2 7 e206 10.1371/journal.pbio.0020206 15252449 PMC449868

[B5] Sharp AJ Locke DP McGrath SD Cheng Z Bailey JA Vallente RU Pertz LM Clark RA Schwartz S Segraves R Oseroff VV Albertson DG Pinkel D Eichler EE Segmental duplications and copy-number variation in the human genome Am J Hum Genet 2005 77 1 78 88 10.1086/431652 15918152 PMC1226196

[B6] de Smith AJ Walters RG Froguel P Blakemore AI Human genes involved in copy number variation: mechanisms of origin, functional effects and implications for disease Cytogenet Genome Res 2008 123 1-4 17 26 10.1159/000184688 19287135 PMC2920180

[B7] Hastings PJ Lupski JR Rosenberg SM Ira G Mechanisms of change in gene copy number Nat Rev Genet 2009 10 8 551 64 10.1038/nrg2593 19597530 PMC2864001

[B8] Otto SP Whitton J Polyploid incidence and evolution Annu Rev Genet 2000 34 401 437 10.1146/annurev.genet.34.1.401 11092833

[B9] The French-Italian Public Consortium for the grapevine genome characterization The grapevine genome sequence suggests ancestral hexaploidization in major angiosperm phyla Nature 2007 449 27 463 468 17721507 10.1038/nature06148

[B10] Tang H Wang X Bowers JE Ming R Alam M Paterson AH Unraveling ancient hexaploidy through multiply-aligned angiosperm gene maps Genome Res 2008 18 12 1944 1954 10.1101/gr.080978.108 18832442 PMC2593578

[B11] Cannon SB Mitra A Baumgarten A Young ND May G The roles of segmental and tandem gene duplication in the evolution of large gene families in *Arabidopsis thaliana* BMC Plant Biol 2004 4 10 10.1186/1471-2229-4-10 15171794 PMC446195

[B12] Park S Oh S Han KH Large-scale computational analysis of poplar ESTs reveals the repertoire and unique features of expressed genes in the poplar genome Mol Breeding 2004 14 4 429 440 10.1007/s11032-004-0603-x

[B13] Lin H Ouyang S Egan A Nobuta K Haas BJ Zhu W Gu X Silva JC Meyers BC Buell CR Characterization of paralogous protein families in rice BMC Plant Biol 2008 8 18 10.1186/1471-2229-8-18 18284697 PMC2275729

[B14] Johnson DA Hill JP Thomas MA The monosaccharide transporter gene family in land plants is ancient and shows differential subfamily expression and expansion across lineages BMC Evol Biol 2006 6 64 21 16923188 10.1186/1471-2148-6-64 PMC1578591

[B15] Gingerich DJ Hanada K Shiu SH Large-scale, lineage-specific expansion of a bric-a-brac/tramtrack/broad complex ubiquitin-ligase gene family in rice Plant Cell 2007 19 8 2329 2348 10.1105/tpc.107.051300 17720868 PMC2002615

[B16] Hanada K Zou C Lehti-Shiu MD Shinozaki K Shiu SH Importance of lineage-specific expansion of plant tandem duplicates in the adaptive response to environmental stimuli Plant Physiol 2008 148 2 993 1003 10.1104/pp.108.122457 18715958 PMC2556807

[B17] Wagner A The fate of duplicated genes: loss or new function? BioEssays 1998 20 10 785 788 10.1002/(SICI)1521-1878(199810)20:10<785::AID-BIES2>3.0.CO;2-M 10200118

[B18] Maere S De Bodt S Raes J Casneuf T Van Montagu M Kuiper M Peer Y Van de Modeling gene and genome duplications in eukaryotes Proc Natl Acad Sci USA 2005 102 15 5454 5459 10.1073/pnas.0501102102 15800040 PMC556253

[B19] Barker MS Kane NC Matvienko M Kozik A Michelmore RW Knapp SJ Rieserberg LH Multiple paleopolyploidizations during the evolution of the Compositae reveal parallel patterns of duplicate gene retention after millions of years Mol Biol Evol 2008 25 11 2445 2455 10.1093/molbev/msn187 18728074 PMC2727391

[B20] Gu Z Nicolae D Lu HH Li WH Rapid divergence in expression between duplicated gene inferred from microarray data Trends Genet 2002 18 12 609 613 10.1016/S0168-9525(02)02837-8 12446139

[B21] Rastogi S Liberles DA Subfunctionalization of duplicated genes as a transition state to neofunctionalization BMC Evol Biol 2005 5 1 28 10.1186/1471-2148-5-28 15831095 PMC1112588

[B22] Perry GH Dominy NJ Claw KG Lee AS Fiegler H Redon R Werner J Villanea FA Mountain JL Misra R Carter NP Lee C Stone AC Diet and the evolution of human amylase gene copy number variation Nat Genet 2007 39 10 1256 1260 10.1038/ng2123 17828263 PMC2377015

[B23] Sax K The origin of the Pomoideae Proc Amer Soc Hort Sci 1933 30 147 150

[B24] Evans RC Campbell CS The origin of the apple subfamily (Maloideae; Rosaceae) is clarified by DNA sequence data from duplicated GBSSI genes Am J Bot 2002 89 9 1478 1484 10.3732/ajb.89.9.1478 21665749

[B25] Blanc G Wolfe KH Widespread paleopolyploidy in model plant species inferred from age distributions of duplicated genes Plant Cell 2004 16 7 1667 1678 10.1105/tpc.021345 15208399 PMC514152

[B26] Schlueter JA Dixon P Granger C Grant D Clark L Doyle JJ Shoemaker RC Mining EST databases to resolve evolutionary events in major crop species Genome 2004 47 5 868 876 10.1139/g04-047 15499401

[B27] Cui L Wall PK Leebens-Mack JH Lindsay BG Soltis DE Doyle JJ Soltis PS Carlson JE Arumuganathan K Barakat A Albert VA Ma H dePamphilis CW Widespread genome duplications throughout the history of flowering plants Genome Res 2006 16 6 738 749 10.1101/gr.4825606 16702410 PMC1479859

[B28] Genome Database for Rosaceae http://www.bioinfo.wsu.edu/gdr/

[B29] Newcomb RD Crowhurst RN Gleave AP Rikkerink EHA Allan AC Beuning LL Bowen JH Gera E Jamieson KR Janssen BJ Laing WA McArtney S Nain B Ross GS Snowden KC Souleyre EJF Walton EF Yauk YK Analyses of expressed sequence tags from apple Plant Physiol 2006 141 1 147 166 10.1104/pp.105.076208 16531485 PMC1459330

[B30] Gasic K Gonzalez DO Thimmapuram J Liu L Malnoy M Gong G Han Y Vodkin LO Aldwinckle HS Carroll NJ Orvis KS Goldsbrough P Clifton S Pape D Fulton L Martin J Theising B Wisniewski ME Fazio G Feltus FA Korban SS Comparative analysis and functional annotation of a large expressed sequence tag collection of apple Plant Genome 2009 2 1 23 38 10.3835/plantgenome2008.11.0014

[B31] Park S Sugimoto N Larson MD Beaudry R van Nocker S Identification of genes with potential roles in apple fruit development and biochemistry through large-scale statistical analysis of expressed sequence tags Plant Physiol 2006 141 3 811 824 10.1104/pp.106.080994 16825339 PMC1489918

[B32] Audic S Claverie JM The significance of digital gene expression profiles Genome Res 1997 7 10 986 995 9331369 10.1101/gr.7.10.986

[B33] Chevreau E Lespinasse Y Gallet M Inheritance of pollen enzymes and polyploid origin of apple (*Malus *× *domestica *Borkh.) Theor Appl Genet 1985 71 2 268 277 10.1007/BF00252066 24247393

[B34] Lynch M Conery JS The evolutionary fate and consequences of duplicate genes Science 2000 290 5494 1151 1155 10.1126/science.290.5494.1151 11073452

[B35] Koch MA Haubold B Mitchell-Olds T Comparative evolutionary analysis of chalcone synthase and alcohol dehydrogenase loci in *Arabidopsis*, *Arabis*, and related genera (Brassicaceae) Mol Biol Evol 2000 17 10 1483 1498 11018155 10.1093/oxfordjournals.molbev.a026248

[B36] DeVore ML Pigg KB A brief review of the fossil history of the family Rosaceae with a focus on the Eocene Okanogan Highlands of eastern Washington State, USA, and British Columbia, Canada Plant Syst Evol 2007 266 1-2 45 57 10.1007/s00606-007-0540-3

[B37] Soria-Hernanz DF Fiz-Palacios O Braverman JM Hamilton MB Reconsidering the generation time hypothesis based on nuclear ribosomal *ITS *sequence comparisons in annual and perennial angiosperms BMC Evol Biol 2008 8 344 10.1186/1471-2148-8-344 19113991 PMC2637270

[B38] Tuskan GA Difazio S Jansson S Bohlmann J Grigoriev I Hellsten U Putnam N Ralph S Rombauts S Salamov A Schein J Sterck L Aerts A Bhalerao RR Bhalerao RP Blaudez D Boerjan W Brun A Brunner A Busov V Campbell M Carlson J Chalot M Chapman J Chen GL Cooper D Coutinho PM Couturier J Covert S Cronk Q Cunningham R Davis J Degroeve S Déjardin A Depamphilis C Detter J Dirks B Dubchak I Duplessis S Ehlting J Ellis B Gendler K Goodstein D Gribskov M Grimwood J Groover A Gunter L Hamberger B Heinze B Helariutta Y Henrissat B Holligan D Holt R Huang W Islam-Faridi N Jones S Jones-Rhoades M Jorgensen R Joshi C Kangasjärvi J Karlsson J Kelleher C Kirkpatrick R Kirst M Kohler A Kalluri U Larimer F Leebens-Mack J Leplé JC Locascio P Lou Y Lucas S Martin F Montanini B Napoli C Nelson DR Nelson C Nieminen K Nilsson O Pereda V Peter G Philippe R Pilate G Poliakov A Razumovskaya J Richardson P Rinaldi C Ritland K Rouzé P Ryaboy D Schmutz J Schrader J Segerman B Shin H Siddiqui A Sterky F Terry A Tsai CJ Uberbacher E Unneberg P Vahala J Wall K Wessler S Yang G Yin T Douglas C Marra M Sandberg G Peer Y Van de Rokhsar D The genome of black cottonwood, *Populus trichocarpa *(Torr. & Gray) Science 2006 313 5793 1596 1604 10.1126/science.1128691 16973872

[B39] Soltis DE Albert VA Leebens-Mack J Bell CD Paterson AH Zheng C Sankoff D dePamphilis CW Wall PK Soltis PS Polyploidy and angiosperm diversification Am J Bot 2009 96 1 336 348 10.3732/ajb.0800079 21628192

[B40] Lyons E Pedersen B Kane J Alam M Ming R Tang H Wang X Bowers J Paterson A Lisch D Freeling M Finding and comparing syntenic regions among Arabidopsis and the outgroups papaya, poplar, and grape: CoGe with rosids Plant Physiol 2008 148 4 1772 1781 10.1104/pp.108.124867 18952863 PMC2593677

[B41] Zhang L Vision TJ Gaut BS Patterns of nucleotide substitution among simultaneously duplicated gene pairs in *Arabidopsis thaliana* Mol Biol Evol 2002 19 9 1464 1473 12200474 10.1093/oxfordjournals.molbev.a004209

[B42] Senchina DS Alvarez I Cronn RC Liu B Rong J Noyes RD Paterson AH Wing RA Wilkins TA Wendel JF Rate variation among nuclear genes and the age of polyploidy in *Gossypium* Mol Biol Evol 2003 20 4 633 643 10.1093/molbev/msg065 12679546

[B43] Huynen MA van Nimwegen E The frequency distribution of gene family sizes in complete genomes Mol Biol Evol 1998 15 5 583 589 9580988 10.1093/oxfordjournals.molbev.a025959

[B44] Hughes T Liberles DA The power-law distribution of gene family size is driven by the pseudogenisation rate's heterogeneity between gene families Gene 2008 414 1-2 85 94 10.1016/j.gene.2008.02.014 18378100

[B45] Chothia C Gough J Vogel C Teichmann SA Evolution of the protein repertoire Science 2003 300 5626 1701 1703 10.1126/science.1085371 12805536

[B46] Arabidopsis Genome Initiative Analysis of the genome sequence of the flowering plant *Arabidopsis thaliana* Nature 2000 408 6814 796 815 10.1038/35048692 11130711

[B47] Goff SA Ricke D Lan TH Presting G Wang R Dunn M Glazebrook J Sessions A Oeller P Varma H Hadley D Hutchison D Martin C Katagiri F Lange BM Moughamer T Xia Y Budworth P Zhong J Miguel T Paszkowski U Zhang S Colbert M Sun WL Chen L Cooper B Park S Wood TC Mao L Quail P Wing R Dean R Yu Y Zharkikh A Shen R Sahasrabudhe S Thomas A Cannings R Gutin A Pruss D Reid J Tavtigian S Mitchell J Eldredge G Scholl T Miller RM Bhatnagar S Adey N Rubano T Tusneem N Robinson R Feldhaus J Macalma T Oliphant A Briggs S A draft sequence of the rice genome (*Oryza sativa *L. ssp. japonica) Science 2002 296 5565 92 100 10.1126/science.1068275 11935018

[B48] Shiu SH Bleecker AB Plant receptor-like kinase gene family: diversity, function, and signaling Sci STKE 2001 18 113 RE22 10.1126/stke.2001.113.re22 11752632

[B49] Wrzaczek M Hirt H Plant MAP kinase pathways: how many and what for? Biol Cell 2001 93 1-2 81 87 10.1016/S0248-4900(01)01121-2 11730326

[B50] Coon MJ Cytochrome P450: nature's most versatile biological catalyst Annu Rev Pharmacol Toxicol 2005 45 1 25 10.1146/annurev.pharmtox.45.120403.100030 15832443

[B51] McHale L Tan X Koehl P Michelmore RW Plant NBS-LRR proteins: adaptable guards Genome Biol 2006 7 4 212 10.1186/gb-2006-7-4-212 16677430 PMC1557992

[B52] Freeling M Bias in plant gene content following different sorts of duplication: tandem, whole-genome, segmental, or by transposition Annu Rev Plant Biol 2009 60 433 453 10.1146/annurev.arplant.043008.092122 19575588

[B53] Edger PP Pires JC Gene and genome duplications: the impact of dosage-sensitivity on the fate of nuclear genes Chromosome Res 2009 17 5 699 717 10.1007/s10577-009-9055-9 19802709

[B54] Thomas BC Pedersen B Freeling M Following tetraploidy in an Arabidopsis ancestor, genes were removed preferentially from one homeolog leaving clusters enriched in dose-sensitive genes Genome Res 2006 16 7 934 46 10.1101/gr.4708406 16760422 PMC1484460

[B55] Freeling M Lyons E Pedersen B Alam M Ming R Lisch D Many or most genes in *Arabidopsis *transposed after the origin of the order Brassicales Genome Res 2008 18 12 1924 1937 10.1101/gr.081026.108 18836034 PMC2593585

[B56] Klimmek F Sjödin A Noutsos C Leister D Jansson S Abundantly and rarely expressed Lhc protein genes exhibit distinct regulation patterns in plants Plant Physiol 2006 140 3 793 804 10.1104/pp.105.073304 16524980 PMC1400566

[B57] Oakley RV Wang YS Ramakrishna W Harding SA Tsai CJ Differential expansion and expression of alpha- and beta-tubulin gene families in *Populus* Plant Physiol 2007 145 3 961 973 10.1104/pp.107.107086 17885081 PMC2048781

[B58] Carroll AJ Heazlewood JL Ito J Millar AH Analysis of the *Arabidopsis *cytosolic ribosome proteome provides detailed insights into its components and their post-translational modification Mol Cell Proteomics 2008 7 2 347 69 17934214 10.1074/mcp.M700052-MCP200

[B59] Whittle CA Krochko JE Transcript profiling provides evidence of functional divergence and expression networks among ribosomal protein gene paralogs in *Brassica napus* Plant Cell 2009 21 8 2203 19 10.1105/tpc.109.068411 19706795 PMC2751962

[B60] Braun FN Liberles DA Retention of enzyme gene duplicates by subfunctionalization Int J Biol Macromol 2003 33 1-3 19 22 10.1016/S0141-8130(03)00059-X 14599579

[B61] Blanc G Wolfe KH Functional divergence of duplicated genes formed by polyploidy during *Arabidopsis *evolution Plant Cell 2004 16 7 1647 1659 10.1105/tpc.160710 15208398 PMC514153

[B62] Duarte JM Cui L Wall PK Zhang Q Zhang X Leebens-Mack J Ma H Altman N dePamphilis CW Expression pattern shifts following duplication indicative of subfunctionalization and neofunctionalization in regulatory genes of *Arabidopsis* Mol Biol Evol 2006 23 2 469 478 10.1093/molbev/msj051 16280546

[B63] Shulaev V Korban SS Sosinski B Abbott AG Aldwinckle HS Folta KM Iezzoni A Main D Arús P Dandekar AM Lewers K Brown SK Davis TM Gardiner SE Potter D Veilleux RE Multiple models for Rosaceae genomics Plant Physiol 2008 147 3 985 1003 10.1104/pp.107.115618 18487361 PMC2442536

[B64] Flagel LE Wendel JF Gene duplication and evolutionary novelty in plants New Phytol 2009 183 3 557 564 10.1111/j.1469-8137.2009.02923.x 19555435

[B65] Ni Z Kim ED Ha M Lackey E Liu J Zhang Y Sun Q Chen ZJ Altered circadian rhythms regulate growth vigor in hybrids and allopolyploids Nature 2009 457 7227 327 331 10.1038/nature07523 19029881 PMC2679702

[B66] Tiffney BH The Eocene North Atlantic land bridge: its importance in the Tertiary and modern phytogeography of the northern hemisphere J Arnold Arbor 1985 66 243 263

[B67] Aldasoro JJ Aedo C Navarro C Phylogenetic and phytogeographycal relationships in Maloideae (Rosaceae) based on morphological and anatomical characters Blumea 2005 50 3 32

[B68] Wolfe JA A paleobotanical interpretation of the Tertiary climate in the northern hemisphere American Scientist 66 694 703

[B69] Morin X Améglio T Ahas R Kurz-Besson C Lanta V Lebourgeois F Miglietta F Chuine I Variation in cold hardiness and carbohydrate concentration from dormancy induction to bud burst among provenances of three European oak species Tree Physiol 2007 27 6 817 825 17331900 10.1093/treephys/27.6.817

[B70] Kaplan F Kopka J Sung DY Zhao W Popp M Porat R Guy CL Transcript and metabolite profiling during cold acclimation of *Arabidopsis *reveals an intricate relationship of cold-regulated gene expression with modifications in metabolite content Plant J 2007 50 6 967 981 10.1111/j.1365-313X.2007.03100.x 17461790

[B71] Pagter M Jensen CR Petersen KK Liu F Arora R Changes in carbohydrates, ABA and bark proteins during seasonal cold acclimation and deacclimation in *Hydrangea *species differing in cold hardiness Physiol Plant 2008 134 3 473 485 10.1111/j.1399-3054.2008.01154.x 18636985

[B72] Maruyama K Takeda M Kidokoro S Yamada K Sakuma Y Urano K Fujita M Yoshiwara K Matsukura S Morishita Y Sasaki R Suzuki H Saito K Shibata D Shinozaki K Yamaguchi-Shinozaki K Metabolic pathways involved in cold acclimation identified by integrated analysis of metabolites and transcripts regulated by DREB1A and DREB2A Plant Physiol 2009 150 4 1972 1980 10.1104/pp.109.135327 19502356 PMC2719109

[B73] Kozlowski TT Carbohydrate sources and sinks in woody plants Bot Rev 1992 58 2 107 122 10.1007/BF02858600

[B74] Cunningham SC Read J Comparison of temperate and tropical rainforest tree species: photosynthetic response to growth temperature Oecologia 2002 133 112 119 10.1007/s00442-002-1034-1 28547297

[B75] Fawcett JA Maere S Peer Y Van de Plants with double genomes might have had a better chance to survive the Cretaceous-Tertiary extinction event Proc Natl Acad Sci USA 2009 106 14 5737 5742 10.1073/pnas.0900906106 19325131 PMC2667025

[B76] Peer Y Van de Maere S Meyer A The evolutionary significance of ancient genome duplications Nat Rev Genet 2009 10 10 725 732 10.1038/nrg2600 19652647

[B77] Xiao H Jiang N Schaffner E Stockinger EJ Knaap E van der A retrotransposon-mediated gene duplication underlies morphological variation of tomato fruit Science 2008 319 5869 1527 1530 10.1126/science.1153040 18339939

[B78] Gabaldón T Large-scale assignment of orthology: back to phylogenetics? Genome Biol 2008 9 10 235 10.1186/gb-2008-9-10-235 18983710 PMC2760865

[B79] The Gene Index Project http://compbio.dfci.harvard.edu/tgi/software/

[B80] Quackenbush J Liang F Holt I Pertea G Upton J The TIGR Gene Indices: reconstruction and representation of expressed gene sequences Nucleic Acids Res 2000 28 1 141 145 10.1093/nar/28.1.141 10592205 PMC102391

[B81] Altschul SF Madden TL Schäffer AA Zhang J Zhang Z Miller W Lipman DJ Gapped BLAST and PSI-BLAST: a new generation of protein database search programs Nucleic Acids Res 1997 25 17 3389 3402 10.1093/nar/25.17.3389 9254694 PMC146917

[B82] Birney E Clamp M Durbin R GeneWise and Genomewise Genome Res 2004 14 5 988 995 10.1101/gr.1865504 15123596 PMC479130

[B83] Thompson JD Higgins DG Gibson TJ CLUSTAL W: improving the sensitivity of progressive multiple sequence alignment through sequence weighting, position-specific gap penalties and weight matrix choice Nucleic Acids Res 1994 22 22 4673 4680 10.1093/nar/22.22.4673 7984417 PMC308517

[B84] Goldman N Yang Z A codon-based model of nucleotide substitution for protein-coding DNA sequences Mol Biol Evol 1994 11 5 725 736 7968486 10.1093/oxfordjournals.molbev.a040153

[B85] Yang Z PAML: a program package for phylogenetic analysis by maximum likelihood Comput Appl Biosci 1997 13 5 555 556 9367129 10.1093/bioinformatics/13.5.555

[B86] Tamura K Dudley J Nei M Kumar S MEGA4: Molecular Evolutionary Genetics Analysis (MEGA) software version 4.0 Mol Biol Evol 2007 24 8 1596 1599 10.1093/molbev/msm092 17488738

[B87] Ronquist F Huelsenbeck JP MrBayes 3: Bayesian phylogenetic inference under mixed models Bioinformatics 2003 19 12 1572 4 10.1093/bioinformatics/btg180 12912839

[B88] Page RD TreeView: an application to display phylogenetic trees on personal computers Comput Appl Biosci 1996 12 4 357 358 8902363 10.1093/bioinformatics/12.4.357

[B89] The Gene Ontology http://www.geneontology.org/

[B90] Conesa A Götz S García-Gómez JM Terol J Talón M Robles M Blast2GO: a universal tool for annotation, visualization and analysis in functional genomics research Bioinformatics 2005 21 18 3674 3676 10.1093/bioinformatics/bti610 16081474

[B91] Götz S García-Gómez JM Terol J Williams TD Nagaraj SH Nueda MJ Robles M Talón M Dopazo J Conesa A High-throughput functional annotation and data mining with the Blast2GO suite Nucleic Acids Res 2008 36 10 3420 3435 10.1093/nar/gkn176 18445632 PMC2425479

[B92] Myhre S Tveit H Mollestad T Laegreid A Additional gene ontology structure for improved biological reasoning Bioinformatics 2006 22 16 2020 2027 10.1093/bioinformatics/btl334 16787968

[B93] Martens C Vandepoele K Peer Y Van de Whole-genome analysis reveals molecular innovations and evolutionary transitions in chromalveolate species Proc Natl Acad Sci USA 2008 105 9 3427 3432 10.1073/pnas.0712248105 18299576 PMC2265158

