# Supplementary material for: Dating and functional characterization of duplicated genes in the apple (Malus domestica Borkh.) by analyzing EST data
Source: BMC Plant Biol. 2010 May 14;10:87. doi: 10.1186/1471-2229-10-87 (PMC3095355; doi:10.1186/1471-2229-10-87)

Additional file 21 – AF21_Ks_vs_Pearson-r.pdf

Scatter-plot of *K*s distance (synonymous substitution/synonymous sites) between pairs of duplicated genes, plotted against divergence in gene expression (Pearson's correlation coefficient).

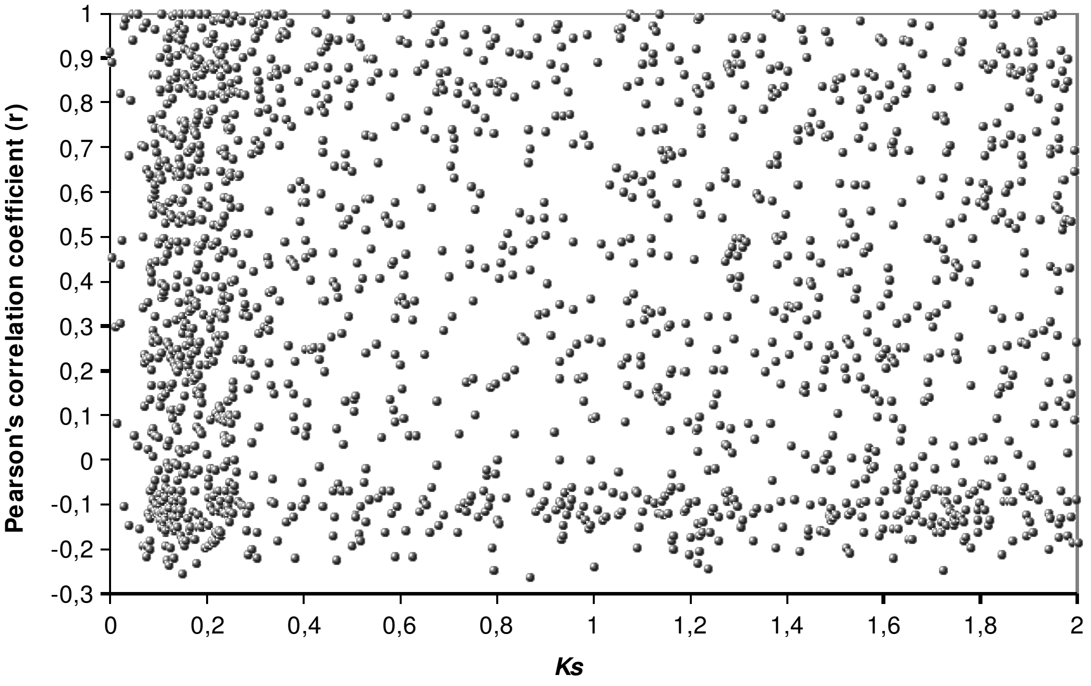

Supplement: Additional file 21 — Scatter-plot of Ks distance (synonymous substitution/synonymous sites) between pairs of duplicated genes, plotted against divergence in gene expression (Pearson's correlation coefficient). [file 1471-2229-10-87-S21.PDF]
