# Supplementary material for: Dating and functional characterization of duplicated genes in the apple (Malus domestica Borkh.) by analyzing EST data
Source: BMC Plant Biol. 2010 May 14;10:87. doi: 10.1186/1471-2229-10-87 (PMC3095355; doi:10.1186/1471-2229-10-87)

Additional file 22 – AF22_FamilySize vs GeneExpresion.pdf
Scatter-plot representing gene family size plotted against gene expression. (A) Individual values represent mean EST counts for each gene family. (B) Individual values represent raw EST counts for each unigene within each gene family.

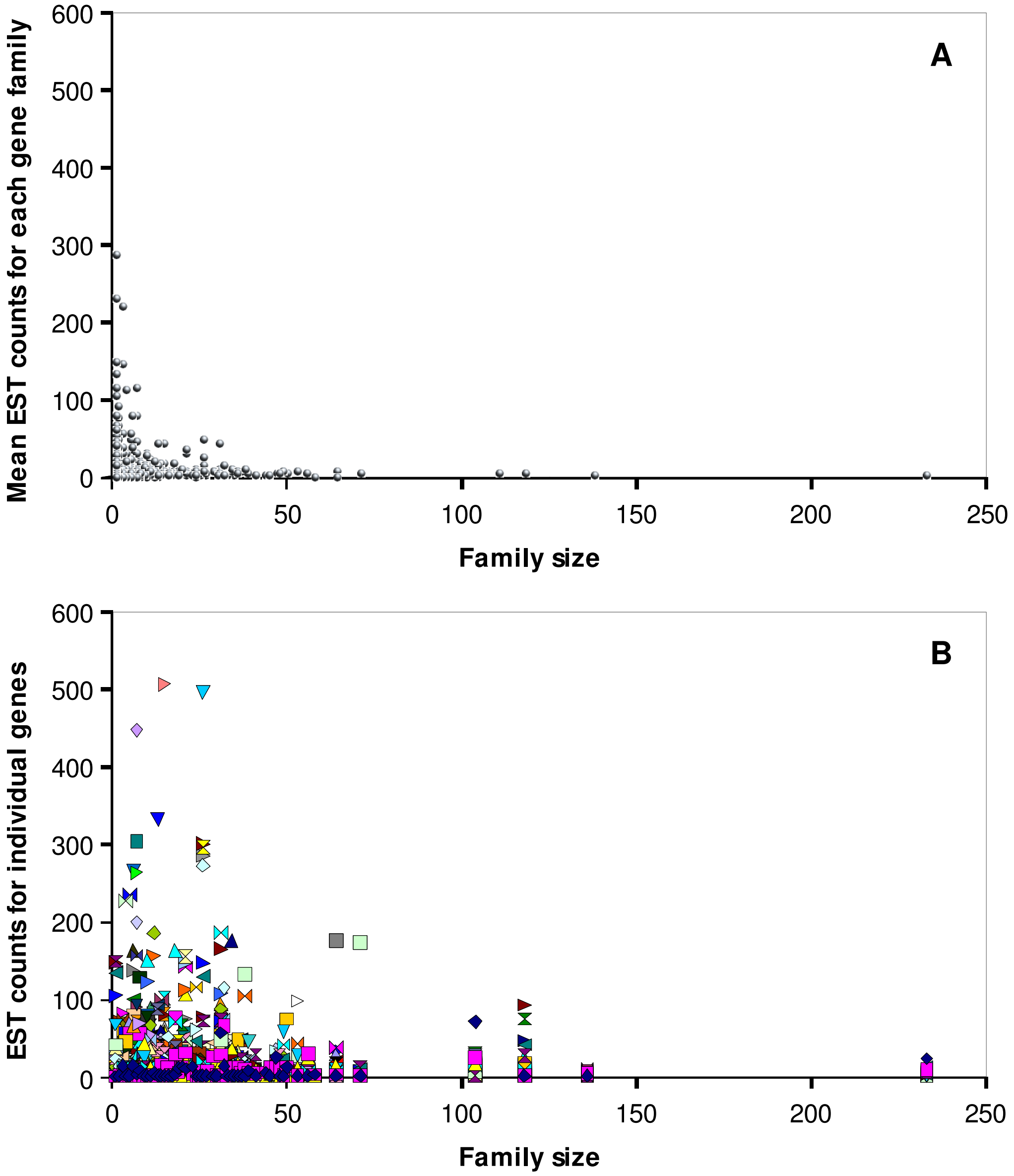

Supplement: Additional file 22 — Scatter-plot representing gene family size plotted against gene expression. (A) Individual values represent mean EST counts for each gene family. (B) Individual values represent raw EST counts for each unigene within each gene family. [file 1471-2229-10-87-S22.PDF]
